# One-Stop Mitral Valve Transcatheter Edge-to-Edge Repair and Left Atrial Appendage Occlusion in Patients with Atrial Fibrillation and Mitral Regurgitation: A Systematic Review and Meta-Analysis

**DOI:** 10.3390/jpm15050197

**Published:** 2025-05-14

**Authors:** Konstantinos Pamporis, Dimitrios Tsiachris, Konstantinos Grigoriou, Paschalis Karakasis, Ioannis Doundoulakis, Panagiotis Theofilis, Panagiotis Kouvatsos, Athanasios Saplaouras, Athanasios Kordalis, Aikaterini-Eleftheria Karanikola, Panagiotis Antonios Goutis, Konstantinos Tsioufis

**Affiliations:** 1 First Cardiology Department, “Hippokration” General Hospital, School of Medicine, National and Kapodistrian University of Athens, 11528 Athens, Greece; dtsiachris@yahoo.com (D.T.); doudougiannis@gmail.com (I.D.); panos.theofilis@hotmail.com (P.T.); panoskouvatsos1@hotmail.com (P.K.); akordalis@gmail.com (A.K.); elinakaranikola@gmail.com (A.-E.K.); ktsioufis@hippocratio.gr (K.T.); 2 Department of Pharmacology, University of Athens, 75 Mikras Asias Avenue, 11527 Goudi, Greece; dinosgrigoriou@gmail.com (K.G.); pangout04@gmail.com (P.A.G.); 3 Second Department of Cardiology, General Hospital “Hippokration”, Aristotle University of Thessaloniki, 54642 Thessaloniki, Greece; pakar15@hotmail.com; 4 Heart Rhythm Management Centre, Postgraduate Program in Cardiac Electrophysiology and Pacing, Universitair Ziekenhuis Brussel, Vrije Universiteit Brussel, European Reference Networks Guard-Heart, 1050 Brussels, Belgium; 5 Arrhythmia Unit, Onassis Cardiac Surgery Center, 17674 Athens, Greece; saplaouras@hotmail.com

**Keywords:** mitral valve transcatheter edge-to-edge repair, mitral regurgitation, left atrial appendage occlusion, atrial fibrillation

## Abstract

**Background/Objectives**: Patients with atrial fibrillation and mitral regurgitation (MR) undergoing transcatheter edge-to-edge mitral valve repair (M-TEER) often have concomitant indications for left atrial appendage occlusion (LAAO), mandating a more personalized treatment approach. This study aimed to examine the effectiveness and safety of combining M-TEER/LAAO in one procedure. **Methods**: MEDLINE (PubMed), Scopus, and Cochrane were searched through 21 March 2025 for studies examining M-TEER/LAAO with or without control (M-TEER only). Double-independent study selection, extraction, and quality assessments were performed. Frequentist random-effects models were used to calculate mean differences (MDs) and risk ratios (RRs) with 95% confidence intervals (CIs). **Results**: Seven studies (223 participants) were included. For M-TEER/LAAO, the mean procedural time was 101.6 min (95% CI = [85.06, 118.13]), the mean radiation time was 29.97 min (95% CI = [23.85, 36.09]), the mean length of stay was 5.21 days (95% CI = [3.31, 7.12]), procedural success was achieved in 89.5% of cases (95% CI = [73.4, 96.3], and post-procedure MR > 2+ occurred in 14.8% of cases (95% CI = [3.6, 44.5]). Compared to M-TEER only, patients with M-TEER/LAAO had similar procedural (RR = 0.91, 95% CI = [0.71, 1.17]) and technical success (RR = 1, 95% CI = [0.94, 1.06]) with a similar risk of acute kidney injury (RR = 1, 95% CI = [0.07, 15.12]), bleeding (RR = 0.40, 95% CI = [0.01, 18.06]), and all-cause death (RR = 0.59, 95% CI = [0.22, 1.54]). M-TEER/LAAO was non-significantly associated with in-hospital death (RR = 3, 95% CI = [0.13, 70.23]), stroke (RR = 3, 95% CI = [0.13, 70.23]), and vascular complications (RR = 1.55, 95% CI = [0.43, 5.59]) compared to M-TEER only. Most patients (34.2%, 95% CI = [2.8, 90.4]) received dual antiplatelet therapy at discharge, followed by anticoagulation only (20.2%, 95% CI = [7.5, 44.3]). **Conclusions**: M-TEER/LAAO can be combined into a single procedure with good peri-procedural outcomes. Safety was also satisfactory; however, some concerns may arise regarding in-hospital death, stroke, and vascular complications. Further research is needed to explore the effectiveness and safety of this combined strategy and elucidate the risk–benefit profile of this personalized treatment approach.

## 1. Introduction

Transcatheter edge-to-edge mitral valve repair (M-TEER) is a minimally invasive treatment option for selected cases of symptomatic patients with severe mitral regurgitation (MR) who are not eligible for surgery. Its safety and effectiveness have been consistently proven in both clinical trials and real-world data, establishing it as the treatment of choice for patients who are inoperable or at high surgical risk for mitral valve replacement (MVR) [1,2,3].

Among the candidates referred for M-TEER, several have concomitant atrial fibrillation (AF), warranting a more personalized treatment strategy. MR is a well-known risk factor for the development of AF, primarily due to the subsequent left atrial enlargement, while AF itself may exacerbate MR by causing mitral annular dilatation [4]. In fact, AF has been associated with adverse outcomes in patients with MR who underwent surgical mitral valve replacement [5,6]. Similar findings have been noted in patients with pre-existing AF undergoing M-TEER, due to significantly higher mortality and an increased risk of major adverse cardiovascular events (MACE) compared to those without AF [7,8]. These findings can partly be attributed to the presence of significant comorbidities in patients with AF, who are often older, more frail, and require lifelong anticoagulation [7]. In a subset of these patients deemed at high bleeding risk or with thromboembolism despite treatment, anticoagulation risks might outweigh benefits. In this group, personalized treatment strategies such as left atrial appendage occlusion (LAAO) may serve as a reliable alternative for stroke prevention. Indeed, despite the widespread use of oral anticoagulation, especially in the elderly [9], it has been shown that LAAO offers comparable protection against thromboembolic events while reducing the risk of clinically relevant bleeding [10].

Considering that both procedures share common interventional steps involving vascular access, echocardiographic/fluoroscopic imaging guidance, and transseptal catheterization to access the left atrium, a combined M-TEER and LAAO seems an attractive approach from both a clinical and logistical perspective. In addition, despite the theoretical procedural challenges of undergoing M-TEER in the presence of AF, primarily due to the irregular rhythm and more dilated mitral annulus, which make leaflet coaptation more difficult, AF had no significant effect on the procedural success rate or total required time, and it was not associated with a higher risk of stroke [7]. Hence, combining M-TEER and LAAO has gained increased attention in recent years, as reflected in the growing number of studies emerging on the topic. This rising interest aligns with the principles of personalized and precision medicine, with the one-step approach tailored to individual patient characteristics. In light of the above, the present systematic review and meta-analysis sought to evaluate the available evidence on the effectiveness and safety of combining M-TEER and LAAO (M-TEER/LAAO) in patients with AF and MR.

## 2. Materials and Methods

The present study was conducted according to the principles outlined in the Cochrane Handbook for systematic reviews of interventions [11], and its reporting followed the Preferred Reporting Items for Systematic Reviews and Meta-Analyses (PRISMA) 2020 guidelines [12] (checklist of reporting items at Table S1). The present study was not pre-registered.

### 2.1. Search Strategy

A double-independent literature search was performed on MEDLINE (via PubMed), Scopus, and the Cochrane Database from inception up to 21 March 2025 without restrictions on language, publication status, or year (full search strategy shown in Table S2). Additional manual searches were performed on clinicaltrials.gov, Epistemonikos, and Google Scholar, while the {citationchaser} R package [13] was used for citation chasing.

### 2.2. Eligibility Criteria

Studies were included based on the following eligibility criteria.

Inclusion criteria:

• Adult patients (≥18 years old) with concomitant indication for M-TEER (moderate-to-severe MR) and LAAO (high bleeding risk, contraindication for use of OAC, recurrent thromboembolism under treatment with OAC).
• Randomized controlled trials (RCTs), observational cohort studies, and case series with a minimum of four patients reporting outcomes on the combined M-TEER/LAAO procedure with or without a control group (M-TEER only).

Exclusion criteria:

• Records that provided data from the same registry (to assure independence of observations).
• Narrative reviews, commentaries, expert opinions, case reports, case-control, and cross-sectional studies.
• Clinical practice guidelines, conference abstracts, protocols, and dissertations.
• Full text not retrievable (authors were contacted, and if a full text was not provided, the record was excluded).

### 2.3. Endpoints

The primary efficacy endpoints of the present study comprised procedural success and technical success, while vascular complications were considered a primary safety endpoint for both analyses (M-TEER/LAAO as a single intervention and M-TEER/LAAO vs M-TEER). Several additional secondary outcomes were explored, including

• Procedure-related outcomes (device success, procedural time, radiation time, volume of administered contrast, number of implanted mitral clips, residual LAAO device leak).
• Short-term clinical outcomes (post-procedure MR > 2+, length of hospitalization, in-hospital death, acute kidney injury, bleeding, hematoma).
• Long-term clinical outcomes (all-cause death, CV mortality, HF hospitalization, stroke, NYHA class reduction, device thrombosis, hematoma).
• Anticoagulation and/or antithrombotic regimen on discharge.

### 2.4. Study Selection

Initially, two independent authors reviewed the titles/abstracts obtained via the aforementioned search strategy. To increase the sensitivity of the study selection process, in case of disagreement at this phase, studies were not excluded. Subsequently, the same authors also independently performed a full-text screening of the selected studies. Disagreements at this final stage were resolved via discussion or consultation with a third author.

### 2.5. Data Extraction

A pilot data extraction form was developed and tested in a subset of three studies. Following discussions, training, and calibration exercises, a standardized extraction form was created for further use. Any disagreements in data extraction were resolved via discussion or consultation with a third and more senior author. For each study, data related to the characteristics (population, intervention, control, outcome, study type, and operator’s experience) and sample sizes of the included studies were collected, as well as demographic (age and sex) and clinical variables (comorbidities, MR type, body mass index, left ventricular ejection fraction, medications, CHA₂DS₂-VASc score, HAS-BLED score, and EuroSCORE II). In case of missing data, study researchers were contacted and/or protocols were scrutinized. The analytical methods implemented to handle data extraction issues were based on the recommendations of the Cochrane Handbook and are presented in Table S3.

### 2.6. Quality Assessment

The methodological quality of the included comparative cohort studies (studies comparing M-TEER/LAAO vs. M-TEER) was thoroughly appraised by two reviewers independently via the Risk of Bias in Non-Randomized Studies of Interventions (ROBINS-I) tool [14]. ROBINS-I constitutes a tool designed to evaluate the risk of bias in observational cohort studies of interventions. It comprises seven domains (confounding, classification of observations, selection of participants, deviations from intended interventions, missing data, measurement of outcome, and selection of the reported result), each rated as a “Low”, “Moderate”, or “High” risk of bias, which together contribute to a conclusion regarding the overall risk of bias of this study. Any discrepancies were addressed through discussion or the involvement of a third reviewer if necessary.

### 2.7. Data Analysis

All analyses were performed using R Statistical Software (v. 4.2). Categorical variables are presented as frequencies with percentages (%), while continuous variables are presented as a mean with standard deviation (SD) when following normal distribution, otherwise as a median with interquartile range (IQR). In the analysis of the procedural characteristics and outcomes of the M-TEER/LAAO as a single intervention group, means were calculated for continuous outcomes and proportions for dichotomous outcomes with 95% confidence intervals (CIs). In the comparative analysis of M-TEER/LAAO vs. M-TEER only, the risk ratio (RR) for dichotomous outcomes and the mean difference (MD) for continuous variables were used. Effect estimates and 95% CIs were calculated using frequentist random-effects Restricted Maximum Likelihood (REML) models. A continuity correction was applied where applicable. The I^2^ was used to estimate the between-study heterogeneity. The values of I^2^ range between 0 and 100, with higher values indicating larger heterogeneity. The proposed I^2^ interpretation cut-offs [15] are 0–40%: might not be important; 30–60%: may represent moderate heterogeneity; 50–90%: may represent substantial heterogeneity; 75–100%: considerable heterogeneity. A subgroup analysis was performed based on the type of each study (observational cohorts vs case series). Further subgroup or sensitivity analyses and investigations for small study effects (including publication bias) were impossible due to the small number of included studies. All results are comprehensively presented in tables and forest plots. For all analyses, a two-tailed *p*-value < 0.05 was considered significant.

## 3. Results

### 3.1. Study Selection and Characteristics of Included Studies

**Figure 1:**
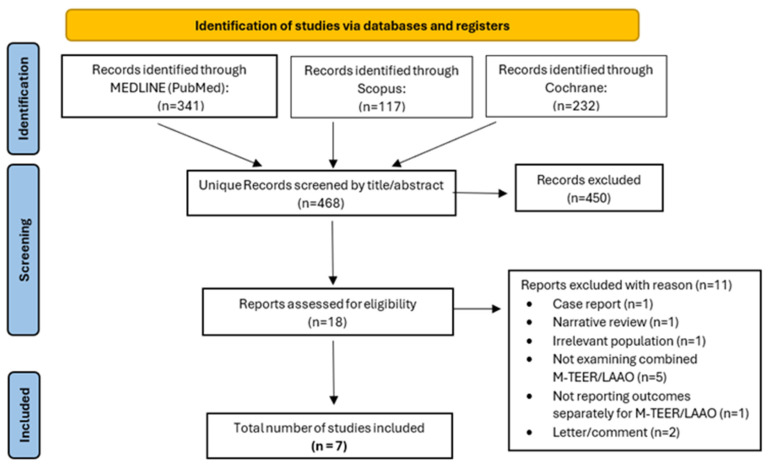
Preferred Reporting Items for Systematic Reviews and Meta-Analyses (PRISMA) flow diagram. M-TEER, mitral valve transcatheter edge-to-edge repair; LAAO, left atrial appendage occlusion.

After 468 unique records were screened, 18 studies were evaluated through full-text screening. Of these, 11 were excluded (table of excluded studies with rationale in Table S4), and finally, 7 studies met the inclusion criteria (Figure 1). The included studies analyzed 233 participants with the following characteristics: mean age of 76.8 (4.5) years, 59% males, 36% coronary artery disease, 93% hypertension, 78% chronic kidney disease, 24% diabetes mellitus, and 19% stroke or transient ischemic attack. From these studies, 5/7 (71%) were observational cohorts (2/5 [40%] with M-TEER as the control group [16,17], and 3/5 [60%] without a control group [18,19,20]), and 2/7 (29%) were case series [21,22] with a minimum of four patients. The operator’s experience was mentioned only in 2/7 (29%) studies, both of which reported a high level of operator experience. The characteristics of the included studies are summarized in Table 1, while patient characteristics are presented in Table 2. Among the two observational cohorts, 1/2 (50%) was of “Low” risk of bias and 1/2 (50%) was of “Moderate” risk of bias based on the ROBINS-I tool (detailed assessment in Table S5).

### 3.2. Single M-TEER/LAAO Intervention Outcomes

The results of the outcomes related only to the combined M-TEER/LAAO procedure (without control) are summarized in Table 3 and in Figures S1–S22. Regarding the primary efficacy outcomes of the combined M-TEER/LAAO, procedural success was achieved in 89.5% (95% CI [73.4, 96.3], I^2^ = 73%, p_heterogeneity_ = 0; 7 studies, 223 participants), and technical success was achieved in 96.2% (95% CI [89.6, 98.7], I^2^ = 0%, p_heterogeneity_ = 0.82; 3 studies, 179 participants). Regarding the primary safety outcome, vascular complications occurred in 8.5% (95% CI [2.8, 23], I^2^ = 43%, p_heterogeneity_ = 0.15; 4 studies, 184 participants) of the patients.

Concerning other procedural aspects of the single M-TEER/LAAO intervention, the mean total procedural time was 101.6 min (95% CI [85.06, 118.13], I^2^ = 92%, p_heterogeneity_ < 0.001; 7 studies, 223 participants), the mean total radiation time was 29.97 min (95% CI [23.85, 36.09], I^2^ = 90%, p_heterogeneity_ < 0.001; 7 studies, 223 participants), the mean total administered contrast was 88.29 mL (95% CI [65.02, 111.56], I^2^ = 81%, p_heterogeneity_ < 0.001; 5 studies, 194 participants), and the mean number of mitral clips placed was 1.64 (95% CI [1.31, 1.97], I^2^ = 91%, p_heterogeneity_ < 0.001; 5 studies, 208 participants). Furthermore, the rate of post-procedure MR > 2+ was 14.8% (95% CI [3.6%, 44.5%], I^2^ = 63%, p_heterogeneity_ = 0.06; 4 studies, 83 participants), while the mean length of hospitalization was 5.21 days (95% CI [3.31, 7.12], I^2^ = 78%, p_heterogeneity_ = 0.004; 4 studies, 177 participants) (Table 3).

With regard to clinical outcomes in patients with M-TEER/LAAO, the rates of all-cause and in-hospital deaths were 11.9% (95% CI [6.9, 19.6], I^2^ = 0%, p_heterogeneity_ = 0.81; 5 studies, 168 participants) and 5.0% (95% CI [1.6, 14.9], I^2^ = 22%, p_heterogeneity_ = 0.28; 4 studies, 183 participants), respectively. Furthermore, the incidence of stroke was 3.5% (95% CI [1.3, 8.9], I^2^ = 0%, p_heterogeneity_ = 0.91; 6 studies, 218 participants), while acute kidney injury occurred in 3% (95% CI [0.7, 11.2], I^2^ = 0%, p_heterogeneity_ = 0.75; 3 studies, 160 participants) of patients. Bleeding complications were observed in 7.5% (95% CI [2.7, 19.5], I^2^ = 49%, p_heterogeneity_ = 0.08; 6 studies, 219 participants). The incidence of HF hospitalization was 9.1% (95% CI [0.6, 63.4], I^2^ = 76%, p_heterogeneity_ = 0.04; 2 studies, 129 participants), the incidence of myocardial infarction was 3.9% (95% CI [0.50, 23.60], I^2^ = 0%, p_heterogeneity_ = 0; 2 studies, 34 participants), and the incidence of NYHA reduction ≥ 1 class was 91.7% (95% CI [37.80, 99.50], 1 study, 5 participants). Finally, most patients were discharged on dual antiplatelet therapy (34%, 95% CI [2.80, 90.40], I^2^ = 94%, p_heterogeneity_ < 0.001; 3 studies, 140 participants), followed by anticoagulation only (20.2%, 95% CI [7.50, 44.30], I^2^ = 70%, p_heterogeneity_ = 0.04; 3 studies, 140 participants). Several other clinical and procedure-related outcomes for the single M-TEER/LAAO procedure are presented in Table 3 and in Figures S1–S22.

### 3.3. Comparative Intervention Outcomes

**Figure 2:**
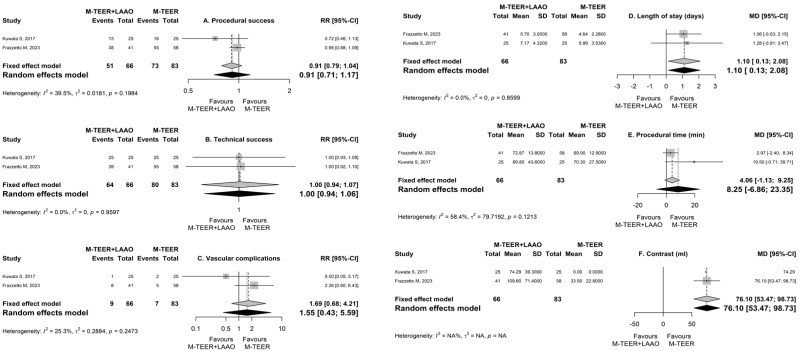
Forest plots for the outcomes of (**A**) procedural success, (**B**) technical success, (**C**) vascular complications, (**D**) length of stay, (**E**) procedural time, and (**F**) administered contrast. RR, risk ratio; MD, mean difference; CI, confidence interval; SD, standard deviation. M-TEER, mitral valve transcatheter edge-to-edge repair; LAAO, left atrial appendage occlusion [16,17].

The results for the comparative intervention outcomes are presented in Table 4, in Figure 2, and in Tables S23–S27. Patients with combined M-TEER/LAAO had similar procedural success (RR = 0.91, 95% CI [0.71, 1.17], *p* = 0.468, I^2^ = 40%, p_heterogeneity_ = 0.20; 2 studies, 149 participants; Figure 2A) and technical success (RR = 1, 95% CI [0.94, 1.06], *p* = 0.966, I^2^ = 0%, p_heterogeneity_ = 0.96; 2 studies, 149 participants; Figure 2B) compared to those who underwent M-TEER only. A non-significant increasing trend was observed in the safety outcome of vascular complications for the M-TEER/LAAO group (RR = 1.55, 95% CI [0.43, 5.59], *p* = 0.503, I^2^ = 25%, p_heterogeneity_ = 0.25; 2 studies, 149 participants; Figure 2C). Patients in the M-TEER/LAAO group had a significantly higher length of stay in the hospital (MD = 1.1 days, 95% CI [0.13, 2.08], *p* = 0.027, I^2^ = 0%, p_heterogeneity_ = 0.86; 2 studies, 149 participants; Figure 2D) and a higher volume of administered contrast (MD = 76.1 mL, 95% CI [53.47, 98.73], *p* < 0.001, I^2^ = Not applicable [N/A], p_heterogeneity_ = N/A; 1 study, 99 participants; Figure 2F). However, there were non-significant differences in the mean total procedural time (Table 4; Figure 2E) and the mean total radiation time (Table 4) between the two procedures.

Regarding safety, there was no statistically significant difference in the relative risk of all-cause death (RR = 0.59, 95% CI [0.22, 1.54], I^2^ = N/A), HF hospitalization (RR = 1.09, 95% CI [0.53, 2.24], *p* = 0.818, I^2^ = N/A, p_heterogeneity_ = N/A; 1 study, 99 participants), bleeding (RR = 0.4, 95% CI [0.01, 18.06], *p* = 0.637, I^2^ = 69%, p_heterogeneity_ = 0.07; 2 studies, 149 participants), or acute kidney injury (RR = 1, 95% CI [0.07, 15.12], *p* = 1, I^2^ = N/A, p_heterogeneity_ = N/A; 2 studies, 149 participants) between the two procedures. However, a trend for an increased risk of in-hospital death (RR = 3, 95% CI [0.13, 70.23], *p* = 0.495, I^2^ = N/A, p_heterogeneity_ = N/A; 2 studies, 149 participants) was observed in patients with combined M-TEER/LAAO compared to controls. Similarly, the risk of stroke tended to be higher in patients with M-TEER/LAAO compared to those with M-TEER only (RR = 3, 95% CI [0.13, 70.23], *p* = 0.495, I^2^ = N/A, p_heterogeneity_ = N/A; 2 studies, 149 participants). Several other clinical and procedure-related comparative outcomes are presented in Table 4.

### 3.4. Subgroup Analysis

The results of the subgroup analysis based on study type (observational cohorts vs case series) for the single M-TEER/LAAO intervention are presented in Table S6. In general, no significant differences between different study types were noted for any outcome.

## 4. Discussion

### 4.1. Summary of Results in the Context of Existing Evidence

The present study constitutes the first systematic review and meta-analysis addressing the effectiveness, safety, and feasibility of a combined M-TEER and LAAO procedure in patients with AF and moderate-to-severe MR. Herein, we demonstrated that this combined, patient-centered, and personalized treatment approach was associated with similar efficacy in peri-procedural outcomes compared to isolated M-TEER in the examined patient groups. However, the small number of included studies, their observational nature, and the limited number of participants render our results prone to confounding, random error, and publication bias, and readers are encouraged to account for these limitations. The one-stop M-TEER-LAAO procedure offers several potential advantages compared to a staged approach. These advantages arise from a more patient-friendly approach and a reduced risk of additional complications associated with staged interventions. Specifically, the combined procedure may reduce the risk of adverse outcomes associated with increased total radiation time, a longer total duration of anticoagulation required during and after the procedure, multiple vascular punctures, repeated anesthesia, and the need for an additional transseptal puncture, which carries the risk of severe complications [23].

The combined M-TEER/LAAO technique achieved high rates of procedural and technical success, with outcomes comparable to those of M-TEER only. Indeed, the procedural success percentage of 89.5% in our study is comparable to the respective ones reported in large M-TEER trials, including MITRA-FR (96%) [24], COAPT (98%) [25], and RESHAPE-HF2 (98.4%) [26]. These findings are partly explained due to the use of the same vascular access and transseptal route, common imaging guidance, and the good maneuverability that the M-TEER sheath provides for the subsequent LAAO [20]. However, there are still concerns and debate regarding key procedural steps in the combined approach, particularly the ideal location of the transseptal puncture and the temporal order of the two procedures. Additionally, the operator’s experience is also a critical aspect to consider, and unfortunately, it was under-reported in the included studies.

Furthermore, the two groups had similarly low rates of post-procedural mitral regurgitation >2+, with no significant differences in hospitalizations for heart failure or all-cause death. In addition, there were no significant differences in the mean total procedural time, mean total radiation time, or mean total administered contrast between the two procedures. Nonetheless, despite peri-procedural outcomes being promising, concerns might be raised due to a possible increase in in-hospital and short-term adverse outcomes. Specifically, the M-TEER/LAAO group had a trend toward greater in-hospital mortality, which may be associated with the baseline risk profile of the enrolled patients and the concomitantly increased in-hospital stay duration. It is well established that prolonged hospitalization predisposes patients to hospital-acquired complications, primarily nosocomial infections [27].

Vascular complications and strokes were also non-significantly increased in the M-TEER/LAAO group. Most vascular accidents were minor in origin (e.g., local hematomas, arteriovenous fistulas), with only rare reports of major incidents (e.g., pseudoaneurysm) [16]. These findings may be explained by the use or not of venous closure, the level of center expertise, and the prolonged manipulation of the femoral vessel, along with the required exchange of sheaths of different sizes. Regarding the non-significantly increased number of strokes in the M-TEER/LAAO group, it is possible that these events are not related to the procedure but rather reflect the high-risk profile of patients requiring both M-TEER and LAAO. Notably, the 3.5% stroke rate of the combined approach encountered in our study is comparable to those reported in large M-TEER trials such as MITRA-FR (4.6%), COAPT (4.4%), and RESHAPE-HF2 (2.2%) [24,25,26], yet slightly higher than the 1.3% reported in the PROTECT-AF trial for LAAO [28]. Regardless, these observations merit further consideration.

Additionally, no significant differences were noted in the risk of acute kidney injury, although the combined strategy required an additional 88.3 mL of contrast. This finding is of significant importance, as it establishes the safety of the combined procedure, given that many patients enrolled in the studies suffer from chronic kidney disease, a recognized risk factor for complications and adverse outcomes following transcatheter interventions [29,30]. Of interest, Armijo G. et al. showed that M-TEER was associated with a 15% incidence of AKI, despite the use of minimal contrast (median volume 50 mL, IQR [37–80]). This suggests that acute kidney injury is primarily caused by complex pathophysiology and patient-related factors, rather than being solely attributed to contrast exposure [31].

Our meta-analysis further suggests that there was no significant difference in bleeding events with the combined approach, despite the high-risk profile of patients for bleeding complications. Hence, the combined M-TEER/LAAO could serve as an integral personalized intervention, even in patients at high bleeding risk. This finding is partly associated with the ability to immediately discontinue oral anticoagulation when adding LAAO, although a higher total dose of heparin is given due to the slightly more prolonged procedure. The incorporation of pharmacokinetic and pharmacogenetic risk factors for major bleeding could further improve outcomes as part of precision-driven and patient-centered decision-making [32]. Finally, there were no significant differences between the two groups regarding cardioembolic events, device failure, or the number of clips required, all of which reflect the efficacy of the M-TEER/LAAO approach.

### 4.2. Implications for Clinical Practice and Future Research

The prevalence of patients with both severe MR and AF is expected to rise as the population ages [33,34]. Due to their comorbidities, these patients often have elevated risks of both thromboembolic and bleeding events, making their treatment with anticoagulation therapy particularly challenging [7]. As demonstrated in our meta-analysis, LAAO combined with M-TEER is a safe, feasible, and effective therapeutic strategy for these cases; therefore, there will likely be a growing demand for this combined procedure in selected patients. Importantly, M-TEER has been associated with reverse atrial remodeling [35], which could lead to a reduced AF burden, thus further limiting the thromboembolic risk in conjunction with LAAO. As previously mentioned, M-TEER and LAAO share common procedural steps, and therefore, the learning curve is expected to be small among interventional cardiologists experienced in either procedure. Additionally, the operator’s experience is an important aspect to consider as a prognostic factor for better outcomes.

Concerning the temporal relationship between interventions, some structural specialists prefer performing M-TEER first to ensure an optimal transseptal site for ideal device positioning [21], while others perform LAAO first, as this approach allows for more efficient use of the sheaths, which theoretically reduces the risk of vascular bleeding [22]. In any case, the sequence of interventions (M-TEER before LAAO) has safety implications, such as avoiding contact with the LAA occluder, which could result in embolization. Furthermore, ideal transseptal puncture sites differ: M-TEER favors a superior–posterior site, while LAAO requires an inferior one. In combined procedures, experienced operators often select a compromise site between these two to accommodate both devices. Hence, since critical technical aspects are not yet fully defined, complications may occur, even in experienced hands.

In addition, M-TEER/LAAO aligns with current trends in healthcare by emphasizing a personalized approach that involves the patient’s perspectives and preferences. Indeed, a recent study demonstrated that the majority of patients with severe MR preferred M-TEER over cardiothoracic surgery, despite being aware of the potentially higher risks of recurrent hospitalizations, reinterventions, and mortality associated with the percutaneous repair [36]. Moreover, some patients may persistently refuse anticoagulant therapy, citing concerns about potential side effects, such as major and minor bleedings, financial constraints, lifestyle adjustments (e.g., unable to consume alcohol), and drug interactions [37]. Patient hesitancy due to a reduced efficacy of oral anticoagulation could also be a potential issue, since it has been shown that LAA thrombi may still be detected in a minority of treated patients [38].

Furthermore, M-TEER/LAAO may offer a feasible alternative for inoperable patients with severe symptomatic MR and AF who are partially or completely resistant to vitamin K antagonists, primarily due to mutations in the vitamin K epoxide reductase complex 1 (VKORC1) gene [39], as well as for those who are non-responders to novel direct oral anticoagulants (DOACs). For the latter group, pharmacogenomic research on DOACs has revealed that genetic variants, such as CES1 and ABCB1 single-nucleotide polymorphisms (SNPs), affect drug levels and may contribute to their therapeutic response [40]. Additional factors, including microRNAs and DNA methylation, could also serve as effect modifiers in the efficacy of DOACs [41]. Incorporating all the above considerations into a precision-based and patient-centered treatment approach could significantly improve outcomes in these patient subgroups.

An important factor that calls into question the widespread adoption of this combined procedure is its reimbursement, as payers typically cover only one procedure at a time in many countries, creating a financial drawback for the treatment institution. In addition, M-TEER/LAAO must demonstrate cost-effectiveness. Given that the majority of patients with severe MR referred for M-TEER are elderly and frail and have significant comorbidities, their life expectancy is short, which limits the potential long-term benefits of LAAO in comparison to the intervention’s cost. To overcome this issue, specific criteria must be established to precisely identify the most suitable patients for concomitant M-TEER and LAAO [42]. In this direction, a thorough screening for AF before M-TEER seems reasonable to better identify suitable patients, especially considering that this population might be at risk for asymptomatic AF manifestation [43,44]. Based on current evidence, it seems reasonable to conclude that the combined procedure is relatively personalized and should be reserved for patients who fulfill the following criteria: (1) inoperable or at high surgical risk; (2) secondary MR ≥ 3+; (3) left ventricular (LV) ejection fraction ≥20% and ≤50%, LV end-systolic diameter ≤ 70 mm, and systolic pulmonary arterial pressure ≤ 70 mm, as determined by the COAPT trial [25]; (3) persistent symptoms despite optimal guideline-directed medical therapy (GDMT), including coronary revascularization and cardiac resynchronization if indicated; (4) clinical AF with a high thromboembolic risk (CHA_2_-DS_2_-VASc score ≥ 2); (5) contraindications for long-term anticoagulant treatment (e.g., previous major bleeding, intracerebral pathology, non-responders to anticoagulants); (6) the absence of significant comorbidities that would result in a life expectancy ≤1 year (e.g., malignancy); and (7) the absence of a left atrial thrombus. Nevertheless, additional research on this subject is required, especially considering the increasing potential of artificial intelligence, from early disease detection to personalized therapy guidance [45]. Several other questions remain open in the field, including the optimal medical therapy upon discharge. Indeed, significant differences and heterogeneous approaches were observed in clinical practice based on the analyzed studies, and this needs to be adequately addressed in the future.

Last but not least, as the use of combined interventional strategies increases, the role of the Heart Team grows in importance. Collaboration across specialties, such as clinical and structural cardiologists, echocardiographers, anesthesiologists, and geriatricians, is essential for further optimizing patient outcomes, improving peri- and post-procedural care, and reducing complication rates. Indeed, multidisciplinary and patient-centered decisions are crucial to provide personalized treatment solutions.

### 4.3. Strengths and Limitations

Among the strengths of our study is the strict adherence to the pertinent methodological and reporting guidelines for the systematic reviews of interventions. Moreover, we have analyzed several procedure- and non-procedure-related outcomes, providing a comprehensive overview regarding the short- and long-term effects of the combined M-TEER/LAAO effects.

Nonetheless, despite the thorough analysis of the currently available data, the observational nature of all included studies significantly limits their inferential potential. Moreover, the number of analyzed studies, as well as the overall number of the included participants, was limited. Additionally, most studies did not have a control group, and the observational cohorts analyzed were of retrospective design. Considerable heterogeneity was also encountered in certain outcomes; nonetheless, the restricted number of included studies limited the potential for further exploration via subgroup analyses, sensitivity analyses, or meta-regression. Additionally, due to the sparsity of the available evidence, cohort studies were pooled with case series, which could further impact the validity of our results and contribute to the increased heterogeneity encountered in certain outcomes. Nevertheless, the subgroup analysis based on the study design did not reveal any significant differences. Finally, the issue of missing data (especially regarding operator experience), coupled with the potential risk of publication bias and selective outcome reporting, may further limit the conclusions of our study. Collectively, our results should be interpreted with caution since they are prone to both confounding and random errors. Hence, the present study should be viewed as hypothesis-generating rather than hypothesis-testing, and readers are encouraged to place more importance on the procedural and short-term outcomes compared to hard clinical outcomes.

### 4.4. Conclusions

In conclusion, M-TEER and LAAO can be combined into a single procedure with good peri-procedural outcomes as part of a personalized treatment approach in selected patient subgroups. In general, safety was also satisfactory; however, some concerns may arise regarding in-hospital death, stroke, and vascular complications. Further research with randomized evidence is needed to explore the effectiveness and safety of this combined strategy and precisely identify appropriate candidates for this one-stop treatment approach.

## Figures and Tables

**Table 1:** Characteristics of included studies.

First Author, Year	Follow-Up (Months)	Population	Intervention	Control	Study Type	Operator’s Experience Level	Number of Participants (n)
Francisco ARG, 2017 [22]	1 (0)	Inoperable severe MR and permanent AF	M-TEER (MitraClip) + LAAO (Watchman)	N/C	Case series	High	5
D’ Amico G, 2021 [18]	15 (15.5)	Severe MR and permanent AF	M-TEER (MitraClip) + LAAO (Amplatzer or Watchman)	N/C	Cohort (prospective)	N/R	30
Kuwata S, 2017 [17]	12	Severe MR and AF	M-TEER (MitraClip) + LAAO (Amplatzer or Amulet)	M-TEER (MitraClip)	Cohort (retrospective)	N/R	50
Tichelbäcker T, 2016 [21]	18 (0)	Severe MR and AF	M-TEER (MitraClip) + LAAO (ProGlide)	N/C	Case series	High	4
Frazzetto M, 2023 [16]	12	Severe MR and AF	M-TEER (MitraClip or PASCAL) + LAAO (Watchman FLX or ProGlide)	M-TEER (MitraClip or PASCAL)	Cohort (retrospective)	N/R	99
Fukuda N, 2024 [19]	12	Moderate-to-severe MR and AF (high bleeding risk, OA contraindication)	M-TEER (MitraClip) + LAAO (Watchman)	N/C	Cohort (retrospective)	N/R	11
Al-Abcha A, 2025 [20]	12	Moderate-to-severe MR and AF	M-TEER (MitraClip or PASCAL) + LAAO (Watchman FLX)	N/C	Cohort (prospective)	N/R	24

Abbreviations: AF, atrial fibrillation; MR, mitral regurgitation; M-TEER, mitral transcatheter edge-to-edge repair; LAAO, left atrial appendage occlusion; N/C, no control; N/R, not reported.

**Table 2:** Population characteristics.

First Author, Year	Number of Participants (n)			Age in Years, Mean (SD)		Males, n (%)		DM, n (%)		CAD, n (%)		MI, n (%)		Permanent AF		Functional/Mixed MR, n (%)		Hypertension, n (%)		CKD, n (%)		LVEF in %, Mean (SD)		CHA_2_DS_2_-VASC Score, Mean (SD)		HAS-BLED Score, Mean (SD)		EuroSCORE II	
	Total	I	C	I	C	I	C	I	C	I	C	I	C	I	C	I	C	I	C	I	C	I	C	I	C	I	C	I	C
Francisco ARG, 2017 [22]	5	5	0	70.8 (8.2)		3 (60)		1 (20)		2 (40)		N/R		5 (100)		5 (100)		5 (100)		5 (100)		26.4 (6)		4.8 (0.8)		3.2 (0.45)		N/R	
D’Amico G, 2021 [18]	30	30	0	74 (9.1)		21 (70)		N/R		N/R		16 (53)		N/R		21 (70)		N/R		22 (73)		42.7 (19.8)		4.4 (0.7)		3.5 (0.8)		8.5 (9.1)	
Kuwata S, 2017 [17]	50	25	25	79.7 (7)	78.8 (8.6)	16 (66)	15 (60)	3 (12)	7 (28)	10 (40)	10 (40)	5 (20)	9 (36)	18 (72)	14 (56)	18 (72)	17 (68)	21 (84)	24 (96)	N/R	N/R	54.1 (12.2)	47.6 (23.2)	4.2 (2)	4 (1.57)	3 (1.57)	3.3 (0.8)	3.3 (2.4)	3.68 (3)
Tichelbäcker T, 2016 [21]	4	4	0	82 (6.48)		2 (50)		N/R		1 (25)		N/R		N/R		3 (75)		N/R		N/R		41.5 (23.2)		5 (0.8)		3.25 (0.5)		N/R	
Frazzetto M, 2023 [16]	99	41	58	77.3 (7.68)	78.3 (6)	24 (58.6)	30 (51.8)	16 (39)	16 (27.5)	20 (48.7)	18 (31)	10 (24.3)	12 (20.6)	N/R	N/R	32 (78)	39 (67.2)	36 (87.8)	52 (89.6)	30 (73.1)	31 (53.4)	43.58 (18.43)	41.1 (17.48)	4.64 (0.76)	4 (1.5)	3.35 (0.76)	2.85 (1.5)	6.79 (2.9)	5.6 (4.3)
Fukuda N, 2024 [19]	11	11	0	79.3 (8.5)		9 (82)		4 (36)		5 (55)		3 (27)		N/R		N/R		2 (18)		N/R		52.1 (11.7)		4.5 (1.7)		2.9 (1.1)		N/R	
Al-Abcha A, 2025 [20]	24	24	0	79.5 (6.3)		20 (83)		N/R		N/R		N/R		8 (32)		18 (75)		24 (100)		19 (79)		45.8 (12.3)		2.8 (1.3)		3.3 (1.5)		N/R	

Abbreviations: I, intervention; C, control; SD, standard deviation; DM, diabetes mellitus; CAD, coronary artery disease; MI, myocardial infarction; AF, atrial fibrillation; MR, mitral regurgitation; CKD, chronic kidney disease; LVEF, left ventricular ejection fraction; N/R, not reported.

**Table 3:** Outcomes related to the single M-TEER/LAAO intervention without a control.

Outcome	Number of Patients	Number of Studies	Point Estimate (95% CI) *	I^2^	p_heterogeneity_
Procedural success (%)	223	7	89.5 (73.40, 96.30)	0.73	0
Technical success (%)	179	3	96.2 (89.60, 98.70)	0	0.82
Vascular complications (%)	184	4	8.50 (2.80, 23)	0.43	0.15
Procedural time (min)	223	7	101.6 (85.06, 118.13)	0.92	<0.001
Radiation time (min)	223	7	29.97 (23.85, 36.09)	0.9	<0.001
Contrast (ml)	194	5	88.29 (65.02, 111.56)	0.81	0.001
Number of clips (n)	208	5	1.64 (1.31, 1.97)	0.91	0.001
Post-procedural MR > 2+ (%)	83	4	14.8 (3.6, 44.5)	0.63	0.06
Length of stay (days)	177	4	5.21 (3.31, 7.12)	0.78	0.004
All-cause death (%)	168	5	11.9 (6.9, 19.6)	0	0.81
In-hospital death (%)	183	4	5 (1.6, 14.9)	0.22	0.28
Stroke (%)	218	6	3.5 (1.3, 8.9)	0	0.91
Acute kidney injury (%)	160	3	3 (0.7, 11.2)	0	0.75
Bleeding (%)	219	6	7.5 (2.7, 19.5)	0.49	0.08
HF hospitalization (%)	129	2	9.1 (0.6, 63.4)	0.76	0.04
Myocardial Infarction (%)	34	2	3.9 (0.5, 23.6)	0	0.35
NYHA reduction ≥ 1 class (%)	5	1	91.7 (37.8, 99.5)	N/A	N/A
Any procedural complication (%)	46	3	7.5 (2.7, 19.5)	0	0.96
CV mortality (%)	30	1	1.6 (0.1, 21.1)	N/A	N/A
LAAO residual leak (%)	139	4	3.7 (1.1, 12)	0	0.79
LAAO device thrombosis (%)	108	3	4.5 (0.9, 19.9)	0	0.48
Embolism (%)	149	5	5.2 (1.4, 17.3)	0.1	0.35
Hematoma (%)	99	1	12.2 (5.2, 26.1)	N/A	N/A
Tamponade (%)	168	5	3.2 (1, 9.4)	0	0.86
DAPT at discharge (%)	140	3	34.2 (2.8, 90.4)	0.94	0.001
SAPT at discharge (%)	140	3	11.7 (1, 63.3)	0.88	0.001
Anticoagulation at discharge (%)	140	3	20.2 (7.5, 44.3)	0.7	0.04
SAPT + anticoagulation at discharge (%)	140	3	11.1 (0.9, 62.3)	0.86	0.001

* Point estimates are expressed in means for continuous outcomes and in proportions for binary outcomes. Abbreviations: CI, confidence interval; N/A, not applicable; NYHA, New York Heart Association; MR, mitral regurgitation; CV, cardiovascular; LAAO, left atrial appendage occlusion; HF, heart failure; SAPT, single antiplatelet therapy; DAPT, dual antiplatelet therapy; SE, systemic embolism.

**Table 4:** Outcomes related to the comparison between M-TEER/LAAO and M-TEER only.

Outcome	Number of Patients	Number of Studies	Point Estimate (95% CI) *	*p*-Value	I^2^	p_heterogeneity_
Procedural success	149	2	0.91 (0.71, 1.17)	0.468	0.4	0.2
Technical success	149	2	1 (0.94, 1.06)	0.966	0	0.96
Vascular complications	149	2	1.55 (0.43, 5.59)	0.503	0.25	0.25
Procedural time (min)	149	2	8.25 (−6.86, 23.35)	0.285	0.58	0.12
Radiation time (min)	149	2	5.1 (−7.77, 17.97)	0.437	0.89	0.002
Contrast (ml)	99	1	76.1 (53.47, 98.73)	<0.001	N/A	N/A
Acute kidney injury	149	2	1 (0.07, 15.12)	1	N/A	N/A
Bleeding	149	2	0.4 (0.01, 18.06)	0.637	0.69	0.07
Number of clips	149	2	0.26 (−0.27, 0.79)	0.328	0.81	0.02
HF hospitalization	99	1	1.09 (0.53, 2.24)	0.818	N/A	N/A
Length of stay (days)	149	2	1.1 (0.13, 2.08)	0.027	0	0.86
In-hospital death	149	2	3 (0.13, 70.23)	0.495	N/A	N/A
All-cause death	99	1	0.59 (0.22, 1.54)	0.282	N/A	N/A
Stroke	149	2	3 (0.13, 70.23)	0.495	N/A	N/A
Post-procedure MR > 2+	50	1	1.67 (0.71, 3.89)	0.237	N/A	N/A
Device success	149	2	0.91 (0.71, 1.17)	0.468	0.4	0.2
Hematoma	99	1	1.77 (0.51, 6.19)	0.372	N/A	N/A

* Point estimates are expressed in mean differences for continuous outcomes and in risk ratios for binary outcomes. Abbreviations: M-TEER, mitral transcatheter edge-to-edge repair; LAAO, left atrial appendage occlusion; CI, confidence interval; N/A, not applicable; HF, heart failure; MR, mitral regurgitation.

## Data Availability

All data extracted and analyzed in this study are available upon reasonable request to the corresponding author.

## Supplementary Materials

The following supporting information can be downloaded at https://www.mdpi.com/article/10.3390/jpm15050197/s1, Figure S1: Forest plot for the outcome of procedural success (M-TEER/LAAO only). CI, confidence interval; Figure S2: Forest plot for the outcome of technical success (M-TEER/LAAO only). CI, confidence interval; Figure S3: Forest plot for the outcome of vascular complications (M-TEER/LAAO only). CI, confidence interval; Figure S4: Forest plot for the outcome of procedural time (M-TEER/LAAO only). CI, confidence interval; Figure S5: Forest plot for the outcome of radiation time (M-TEER/LAAO only). CI, confidence interval; Figure S6: Forest plot for the outcome of administered contrast (M-TEER/LAAO only). CI, confidence interval; Figure S7. Forest plot for the outcome of the number of implanted clips (M-TEER/LAAO only). CI, confidence interval; Figure S8: Forest plot for the outcome of post-procedure MR>2+ (M-TEER/LAAO only). CI, confidence interval; MR, mitral regurgitation; Figure S9: Forest plot for the outcome of the duration of length of stay (M-TEER/LAAO only). CI, confidence interval; Figure S10: Forest plot for the outcome of all-cause death (M-TEER/LAAO only). CI, confidence interval; Figure S11: Forest plot for the outcome of in-hospital death (M-TEER/LAAO only). CI, confidence interval; Figure S12: Forest plot for the outcome of stroke (M-TEER/LAAO only). CI, confidence interval; Figure S13: Forest plot for the outcome of acute kidney injury (M-TEER/LAAO only). CI, confidence inter; Figure S14: Forest plot for the outcome of bleeding (M-TEER/LAAO only). CI, confidence interval; Figure S15: Forest plot for the outcome of HF hospitalization (M-TEER/LAAO only). CI, confidence interval; Figure S16: Forest plot for the outcome of myocardial infarction (M-TEER/LAAO only). CI, confidence interval; Figure S17: Forest plot for the outcome of any procedural complication (M-TEER/LAAO only). CI, confidence interval; Figure S18: Forest plot for the outcome of residual LAAO leak (M-TEER/LAAO only). CI, confidence interval; Figure S19: Forest plot for the outcome of device thrombosis (M-TEER/LAAO only). CI, confidence interval; Figure S20: Forest plot for the outcome of embolism (M-TEER/LAAO only). CI, confidence interval; Figure S21: Forest plot for the outcome of tamponade (M-TEER/LAAO only). CI, confidence interval; Figure S22: Forest plot for the outcome of discharge on dual antiplatelet therapy (DAPT) (M-TEER/LAAO only). CI, confidence interval; Figure S23: Forest plot for the outcome of discharge on single antiplatelet therapy (SAPT) (M-TEER/LAAO only). CI, confidence interval; Figure S24: Forest plot for the outcome of discharge on anticoagulation (M-TEER/LAAO only). CI, confidence interval; Figure S25: Forest plot for the outcome of bleeding (M-TEER/LAAO vs M-TEER). RR, risk ratio; CI, confidence interval; M-TEER, mitral transcatheter edge-to-edge repair; LAAO, left atrial appendage occlusion; Figure S26: Forest plot for the outcome of in-hospital death (M-TEER/LAAO vs M-TEER). RR, risk ratio; CI, confidence interval; M-TEER, mitral transcatheter edge-to-edge repair; LAAO, left atrial appendage occlusion; Figure S27: Forest plot for the outcome of stroke (M-TEER/LAAO vs M-TEER). RR, risk ratio; CI, confidence interval; M-TEER, mitral transcatheter edge-to-edge repair; LAAO, left atrial appendage occlusion; Table S1: Checklist of PRISMA 2020 reported items; Table S2: Search strategy for main databases; Table S3: Approaches to data extraction issue; Table S4: Table of excluded studies with rationale; Table S5: Study quality assessment with ROBINS-I; Table S6: Subgroup analysis based on study type.
