# Supplementary material for: One-Stop Mitral Valve Transcatheter Edge-to-Edge Repair and Left Atrial Appendage Occlusion in Patients with Atrial Fibrillation and Mitral Regurgitation: A Systematic Review and Meta-Analysis
Source: J Pers Med. 2025 May 14;15(5):197. doi: 10.3390/jpm15050197 (PMC12113070; doi:10.3390/jpm15050197)
Supplement: Supplementary file 1 [file jpm-15-00197-s001.zip › jpm-3573623-supplementary.pdf]

SUPPLEMENTAL MATERIAL

OF

One-stop mitral valve transcatheter edge-to-edge repair and left atrial appendage occlusion in patients with atrial fibrillation and mitral regurgitation: a systematic review and meta-analysis

Table of Contents

Supplemental Tables

Table S.1. Checklist of PRISMA 2020 reported items 3

Table S.2. Search strategy for main databases 6

Table S.3. Approaches to data extraction issues 9

Table S.4. Table of excluded studies with rationale. 9

Table S.5. Study quality assessment with ROBINS-I 9

Table S.6. Subgroup analysis based on study type 10

Figure S.1. Forest plot for the outcome of procedural success (M-TEER/LAAO only). 11

Figure S.2. Forest plot for the outcome of technical success (M-TEER/LAAO only). 11

Figure S.3. Forest plot for the outcome of vascular complications (M-TEER/LAAO only). 12

Figure S.4. Forest plot for the outcome of procedural time (M-TEER/LAAO only). 12

Figure S.5. Forest plot for the outcome of radiation time (M-TEER/LAAO only). 13

Figure S.6. Forest plot for the outcome of administered contrast (M-TEER/LAAO only). 14

Figure S.7. Forest plot for the outcome of the number of implanted clips (M-TEER/LAAO only). 14

Figure S.8. Forest plot for the outcome of post-procedure MR>2+ (M-TEER/LAAO only). 15

Figure S.9. Forest plot for the outcome of the duration of length of stay (M-TEER/LAAO only). 15

Figure S.10. Forest plot for the outcome of all-cause death (M-TEER/LAAO only). 16

Figure S.11. Forest plot for the outcome of in-hospital death (M-TEER/LAAO only). 16

Figure S.12. Forest plot for the outcome of stroke (M-TEER/LAAO only). 17

Figure S.13. Forest plot for the outcome of acute kidney injury (M-TEER/LAAO only). 17

Figure S.14. Forest plot for the outcome of bleeding (M-TEER/LAAO only). 18

Figure S.15. Forest plot for the outcome of HF hospitalization (M-TEER/LAAO only). 18

Figure S.16. Forest plot for the outcome of myocardial infarction (M-TEER/LAAO only). 19

Figure S.17. Forest plot for the outcome of any procedural complication (M-TEER/LAAO only). 19

Figure S.18. Forest plot for the outcome of residual LAAO leak (M-TEER/LAAO only). 20

Figure S.19. Forest plot for the outcome of device thrombosis (M-TEER/LAAO only). 20

Figure S.20. Forest plot for the outcome of embolism (M-TEER/LAAO only). 21

Figure S.21. Forest plot for the outcome of tamponade (M-TEER/LAAO only). 21

Figure S.22. Forest plot for the outcome of discharge on dual antiplatelet therapy (DAPT) (M-TEER/LAAO only). 22

Figure S.23. Forest plot for the outcome of discharge on single antiplatelet therapy (SAPT) (M-TEER/LAAO only). 22

Figure S.24. Forest plot for the outcome of discharge on anticoagulation (M-TEER/LAAO only). 22

Figure S.22. Forest plot for the outcome of discharge on single antiplatelet therapy (SAPT) and anticoagulation (M-TEER/LAAO only). 23
Figure S.23. Forest plot for the outcome of radiation time (M-TEER/LAAO vs M-TEER). 23
Figure S.24. Forest plot for the outcome of acute kidney injury (M-TEER/LAAO vs M-TEER). 23
Figure S.25. Forest plot for the outcome of bleeding (M-TEER/LAAO vs M-TEER). 24
Figure S.26. Forest plot for the outcome of in-hospital death (M-TEER/LAAO vs M-TEER). 24
Figure S.27. Forest plot for the outcome of stroke (M-TEER/LAAO vs M-TEER). 24

Table S.1. Checklist of PRISMA 2020 reported items

Section and Topic	Item #	Checklist item	Location (page) where item is reported
**TITLE**			

Section and Topic	Item #	Checklist item	Location (page) where item is reported
Title	1	Identify the report as a systematic review.	1
**ABSTRACT**			
Abstract	2	See the PRISMA 2020 for Abstracts checklist.	3
**INTRODUCTION**			
Rationale	3	Describe the rationale for the review in the context of existing knowledge.	5-6
Objectives	4	Provide an explicit statement of the objective(s) or question(s) the review addresses.	5-6
**METHODS**			
Eligibility criteria	5	Specify the inclusion and exclusion criteria for the review and how studies were grouped for the syntheses.	7
Information sources	6	Specify all databases, registers, websites, organisations, reference lists and other sources searched or consulted to identify studies. Specify the date when each source was last searched or consulted.	6
Search strategy	7	Present the full search strategies for all databases, registers and websites, including any filters and limits used.	Table S.2
Selection process	8	Specify the methods used to decide whether a study met the inclusion criteria of the review, including how many reviewers screened each record and each report retrieved, whether they worked independently, and if applicable, details of automation tools used in the process.	8
Data collection process	9	Specify the methods used to collect data from reports, including how many reviewers collected data from each report, whether they worked independently, any processes for obtaining or confirming data from study investigators, and if applicable, details of automation tools used in the process.	8-9
Data items	10a	List and define all outcomes for which data were sought. Specify whether all results that were compatible with each outcome domain in each study were sought (e.g. for all measures, time points, analyses), and if not, the methods used to decide which results to collect.	7-8
	10b	List and define all other variables for which data were sought (e.g. participant and intervention characteristics, funding sources). Describe any assumptions made about any missing or unclear information.	7-9
Study risk of bias assessment	11	Specify the methods used to assess risk of bias in the included studies, including details of the tool(s) used, how many reviewers assessed each study and whether they worked independently, and if applicable, details of automation tools used in the process.	9
Effect measures	12	Specify for each outcome the effect measure(s) (e.g. risk ratio, mean difference) used in the synthesis or presentation of results.	9-10
Synthesis methods	13a	Describe the processes used to decide which studies were eligible for each synthesis (e.g. tabulating the study intervention characteristics and comparing against the planned groups for each synthesis (item #5)).	9-10
	13b	Describe any methods required to prepare the data for presentation or synthesis, such as handling of missing summary statistics, or data conversions.	N/A
	13c	Describe any methods used to tabulate or visually display results of individual studies and syntheses.	10
	13d	Describe any methods used to synthesize results and provide a rationale for the choice(s). If meta-analysis was performed, describe the model(s), method(s) to identify the presence and extent of statistical heterogeneity, and software package(s) used.	9-10
	13e	Describe any methods used to explore possible causes of heterogeneity among study results (e.g. subgroup analysis, meta-regression).	10
	13f	Describe any sensitivity analyses conducted to assess robustness of the synthesized results.	N/A
Reporting bias assessment	14	Describe any methods used to assess risk of bias due to missing results in a synthesis (arising from reporting biases).	N/A
Certainty assessment	15	Describe any methods used to assess certainty (or confidence) in the body of evidence for an outcome.	N/A
**RESULTS**			
Study selection	16a	Describe the results of the search and selection process, from the number of records identified in the search to the number of studies included in the review, ideally using a flow diagram.	10-11
	16b	Cite studies that might appear to meet the inclusion criteria, but which were excluded, and explain why they were excluded.	Table S.4

Section and Topic	Item #	Checklist item	Location (page) where item is reported
Study characteristics	17	Cite each included study and present its characteristics.	10-11
Risk of bias in studies	18	Present assessments of risk of bias for each included study.	Table S.5
Results of individual studies	19	For all outcomes, present, for each study: (a) summary statistics for each group (where appropriate) and (b) an effect estimate and its precision (e.g. confidence/credible interval), ideally using structured tables or plots.	11-13
Results of syntheses	20a	For each synthesis, briefly summarise the characteristics and risk of bias among contributing studies.	11
	20b	Present results of all statistical syntheses conducted. If meta-analysis was done, present for each the summary estimate and its precision (e.g. confidence/credible interval) and measures of statistical heterogeneity. If comparing groups, describe the direction of the effect.	11-13
	20c	Present results of all investigations of possible causes of heterogeneity among study results.	13
	20d	Present results of all sensitivity analyses conducted to assess the robustness of the synthesized results.	N/A
Reporting biases	21	Present assessments of risk of bias due to missing results (arising from reporting biases) for each synthesis assessed.	N/A
Certainty of evidence	22	Present assessments of certainty (or confidence) in the body of evidence for each outcome assessed.	N/A
**DISCUSSION**			
Discussion	23a	Provide a general interpretation of the results in the context of other evidence.	14-16
	23b	Discuss any limitations of the evidence included in the review.	18
	23c	Discuss any limitations of the review processes used.	18
	23d	Discuss implications of the results for practice, policy, and future research.	16-17
**OTHER INFORMATION**			
Registration and protocol	24a	Provide registration information for the review, including register name and registration number, or state that the review was not registered.	N/A
	24b	Indicate where the review protocol can be accessed, or state that a protocol was not prepared.	N/A
	24c	Describe and explain any amendments to information provided at registration or in the protocol.	N/A
Support	25	Describe sources of financial or non-financial support for the review, and the role of the funders or sponsors in the review.	19
Competing interests	26	Declare any competing interests of review authors.	19
Availability of data, code and other materials	27	Report which of the following are publicly available and where they can be found: template data collection forms; data extracted from included studies; data used for all analyses; analytic code; any other materials used in the review.	19

Abbreviations: PRISMA, Preferred Reporting Items for Systematic Reviews and Meta-Analyses; N/A, not applicable.

Table S.2. Search strategy for main databases

**MEDLINE (via Pubmed)**
("Atrial Fibrillation"[MeSH] OR "atrial fibrillation*"[All Fields] OR "auricular fibrillation*"[All Fields] OR "fibrillation atr*"[All Fields] OR "fibrillation of atr*"[All Fields] OR "fibrillation of the atr*"[All Fields])<br>**AND**<br>("Transcatheter Edge-to-Edge Mitral Valve Repair*"[All Fields] OR "Transcatheter Edge to Edge Mitral Valve Repair*"[All Fields] OR "Transcatheter Edge-to-Edge MVR*"[All Fields] OR "Transcatheter Edge to Edge MVR*"[All Fields] OR "Mitral valve Transcatheter Edge-to-Edge Repair*"[All Fields] OR "Mitral valve Transcatheter Edge to Edge Repair*"[All Fields] OR "MV Transcatheter Edge-to-Edge Repair*"[All Fields] OR "MV Transcatheter Edge to Edge Repair*"[All Fields] OR "Transcatheter Edge-to-Edge MVR*"[All Fields] OR "Transcatheter Edge to Edge MVR*"[All Fields] OR "MVR Transcatheter Edge-to-Edge*"[All Fields] OR "MVR Transcatheter Edge to Edge*"[All Fields] OR "Transcatheter Edge-to-Edge MVR*"[All Fields] OR "Transcatheter Edge to Edge MVR*"[All Fields] OR "TEER"[All Fields] OR "M TEER"[All Fields] OR "M-TEER"[All Fields] OR "MV TEER"[All Fields] OR "Transcatheter mitral valve repair*"[All Fields] OR "Transcatheter MV repair*"[All Fields] OR "Edge-to-edge repair*"[All Fields] OR "Edge-to-edge MV repair*"[All Fields] OR "Edge-to-edge mitral valve repair*"[All Fields] OR "Percutaneous mitral valve repair*"[All Fields] OR "Percutaneous MV repair*"[All Fields] OR "Transcatheter mitral valve intervention*"[All Fields] OR "Transcatheter MV intervention*"[All Fields] OR "TMVI*"[All Fields] OR "Specific devices and procedures*"[All Fields] OR "Transcatheter leaflet coaptation*"[All Fields] OR "Transcatheter MV leaflet coaptation*"[All Fields] OR "Transcatheter mitral leaflet coaptation*"[All Fields] OR "Transcatheter mitral valve leaflet coaptation*"[All Fields] OR "Transcatheter leaflets coaptation*"[All Fields] OR "Transcatheter MV leaflets coaptation*"[All Fields] OR "Transcatheter mitral leaflets coaptation*"[All Fields] OR "Transcatheter mitral valve leaflets coaptation*"[All Fields] OR "Transcatheter cusp coaptation*"[All Fields] OR "Transcatheter MV cusp coaptation*"[All Fields] OR "Transcatheter mitral cusp coaptation*"[All Fields] OR "Transcatheter mitral valve cusp coaptation*"[All Fields] OR "Transcatheter cusps coaptation*"[All Fields] OR "Transcatheter MV cusps coaptation*"[All Fields] OR "Transcatheter mitral cusps coaptation*"[All Fields] OR "Transcatheter mitral valve cusps coaptation*"[All Fields] OR "Endovascular mitral repair*"[All Fields] OR "Endovascular mitral valve repair*"[All Fields] OR "Endovascular MV repair*"[All Fields] OR "MitraClip*"[All Fields] OR "Mitra-Clip*"[All Fields] OR "Mitra Clip*"[All Fields] OR "Mitral Clip*"[All Fields] OR "M-Clip*"[All Fields] OR "PASCAL device*"[All Fields] OR "PASCAL*"[All Fields] OR "PASCAL precision system*"[All Fields] OR "Transcatheter mitral cusp approximation*"[All Fields] OR "Transcatheter mitral valve cusp approximation*"[All Fields] OR "Transcatheter MV cusp approximation*"[All Fields] OR "Transcatheter mitral cusps approximation*"[All Fields] OR "Transcatheter mitral valve cusps approximation*"[All Fields] OR "Transcatheter MV cusps approximation*"[All Fields])<br>**AND**<br>("Left Atrial Appendage Closure"[Mesh] OR "Left Atrial Appendage Closure*"[All Fields] OR "Left atrium Appendage Closure*"[All Fields] OR "LA Appendage Closure*"[All Fields] OR "LAA Closure*"[All Fields] OR "LAAC*"[All Fields] OR "Closure, LAA*"[All Fields] OR "Closure

LAA*"[All Fields] OR "LAA Closure*"[All Fields] OR "Left Atrial Appendage Occlusion*"[All Fields] OR "Left atrium Appendage Occlusion*"[All Fields] OR "LA Appendage Occlusion*"[All Fields] OR "LAA Occlusion*"[All Fields] OR "LAAO*"[All Fields] OR "Left Atrial Appendage Exclusion*"[All Fields] OR "Left atrium Appendage Exclusion*"[All Fields] OR "LA Appendage Exclusion*"[All Fields] OR "LAA Exclusion*"[All Fields] OR "LAAE*"[All Fields] OR "Left Atrial Appendage Ligation*"[All Fields] OR "Left atrium Appendage Ligation*"[All Fields] OR "LA Appendage Ligation*"[All Fields] OR "LAA Ligation*"[All Fields] OR "LAAL*"[All Fields] OR "Watchman Device*"[All Fields] OR "Watchman*"[All Fields] OR "Watchman FLX*"[All Fields] OR "Watchman 2.5*"[All Fields] OR "Amplatzer Amulet*"[All Fields] OR "Amplatzer*"[All Fields] OR "LARIAT Suture*"[All Fields] OR "CLOSURE Device*"[All Fields] OR "LAA Closure Device*"[All Fields] OR "ACURATE LAA*"[All Fields] OR "Atriclip*"[All Fields])
**Scopus**
(TITLE-ABS-KEY("atrial fibrillation*") OR TITLE-ABS-KEY("auricular fibrillation*") OR TITLE-ABS-KEY("fibrillation atr*") OR TITLE-ABS-KEY("fibrillation of atr*") OR TITLE-ABS-KEY("fibrillation of the atr*")) AND (TITLE-ABS-KEY("Transcatheter Edge-to-Edge Mitral Valve Repair*") OR TITLE-ABS-KEY("Transcatheter Edge to Edge Mitral Valve Repair*") OR TITLE-ABS-KEY("Transcatheter Edge-to-Edge MVR*") OR TITLE-ABS-KEY("Transcatheter Edge to Edge MVR*") OR TITLE-ABS-KEY("Mitral valve Transcatheter Edge-to-Edge Repair*") OR TITLE-ABS-KEY("Mitral valve Transcatheter Edge to Edge Repair*") OR TITLE-ABS-KEY("MV Transcatheter Edge-to-Edge Repair*") OR TITLE-ABS-KEY("MV Transcatheter Edge to Edge Repair*") OR TITLE-ABS-KEY("Transcatheter Edge-to-Edge MVR*") OR TITLE-ABS-KEY("Transcatheter Edge to Edge MVR*") OR TITLE-ABS-KEY("MVR Transcatheter Edge-to-Edge*") OR TITLE-ABS-KEY("MVR Transcatheter Edge to Edge*") OR TITLE-ABS-KEY("TEER") OR TITLE-ABS-KEY("M TEER") OR TITLE-ABS-KEY("M-TEER") OR TITLE-ABS-KEY("MV TEER") OR TITLE-ABS-KEY("Transcatheter mitral valve repair*") OR TITLE-ABS-KEY("Transcatheter MV repair*") OR TITLE-ABS-KEY("Edge-to-edge repair*") OR TITLE-ABS-KEY("Edge-to-edge MV repair*") OR TITLE-ABS-KEY("Edge-to-edge mitral valve repair*") OR TITLE-ABS-KEY("Percutaneous mitral valve repair*") OR TITLE-ABS-KEY("Percutaneous MV repair*") OR TITLE-ABS-KEY("Transcatheter mitral valve intervention*") OR TITLE-ABS-KEY("Transcatheter MV intervention*") OR TITLE-ABS-KEY("TMVI*") OR TITLE-ABS-KEY("Specific devices and procedures*") OR TITLE-ABS-KEY("Transcatheter leaflet coaptation*") OR TITLE-ABS-KEY("Transcatheter MV leaflet coaptation*") OR TITLE-ABS-KEY("Transcatheter mitral leaflet coaptation*") OR TITLE-ABS-KEY("Transcatheter mitral valve leaflet coaptation*") OR TITLE-ABS-KEY("Transcatheter leaflets coaptation*") OR TITLE-ABS-KEY("Transcatheter MV leaflets coaptation*") OR TITLE-ABS-KEY("Transcatheter mitral leaflets coaptation*") OR TITLE-ABS-KEY("Transcatheter mitral valve leaflets coaptation*") OR TITLE-ABS-KEY("Transcatheter cusp coaptation*") OR TITLE-ABS-KEY("Transcatheter MV cusp coaptation*") OR TITLE-ABS-KEY("Transcatheter mitral cusp coaptation*") OR TITLE-ABS-KEY("Transcatheter mitral valve cusp coaptation*") OR TITLE-ABS-KEY("Transcatheter cusps coaptation*") OR TITLE-ABS-KEY("Transcatheter MV cusps coaptation*") OR TITLE-ABS-KEY("Transcatheter mitral cusps coaptation*") OR TITLE-ABS-KEY("Transcatheter mitral valve cusps coaptation*") OR TITLE-ABS-KEY("Endovascular mitral repair*") OR TITLE-ABS-KEY("Endovascular mitral valve repair*") OR TITLE-ABS-KEY("Endovascular MV repair*") OR TITLE-ABS-KEY("MitraClip*") OR TITLE-ABS-KEY("Mitra-Clip*") OR TITLE-ABS-KEY("Mitra Clip*") OR TITLE-ABS-KEY("Mitral Clip*") OR TITLE-ABS-

KEY("M-Clip*") OR TITLE-ABS-KEY("PASCAL device*") OR TITLE-ABS-KEY("PASCAL*") OR TITLE-ABS-KEY("PASCAL precision system*") OR TITLE-ABS-KEY("Transcatheter mitral cusp approximation*") OR TITLE-ABS-KEY("Transcatheter mitral valve cusp approximation*") OR TITLE-ABS-KEY("Transcatheter MV cusp approximation*") OR TITLE-ABS-KEY("Transcatheter mitral cusps approximation*") OR TITLE-ABS-KEY("Transcatheter mitral valve cusps approximation*") OR TITLE-ABS-KEY("Transcatheter MV cusps approximation*")) AND (TITLE-ABS-KEY("Left Atrial Appendage Closure*") OR TITLE-ABS-KEY("Left atrium Appendage Closure*") OR TITLE-ABS-KEY("LA Appendage Closure*") OR TITLE-ABS-KEY("LAA Closure*") OR TITLE-ABS-KEY("LAAC*") OR TITLE-ABS-KEY("Closure, LAA*") OR TITLE-ABS-KEY("Closure LAA*") OR TITLE-ABS-KEY("LAA Closure*") OR TITLE-ABS-KEY("Left Atrial Appendage Occlusion*") OR TITLE-ABS-KEY("Left atrium Appendage Occlusion*") OR TITLE-ABS-KEY("LA Appendage Occlusion*") OR TITLE-ABS-KEY("LAA Occlusion*") OR TITLE-ABS-KEY("LAAO*") OR TITLE-ABS-KEY("Left Atrial Appendage Exclusion*") OR TITLE-ABS-KEY("Left atrium Appendage Exclusion*") OR TITLE-ABS-KEY("LA Appendage Exclusion*") OR TITLE-ABS-KEY("LAA Exclusion*") OR TITLE-ABS-KEY("LAAE*") OR TITLE-ABS-KEY("Left Atrial Appendage Ligation*") OR TITLE-ABS-KEY("Left atrium Appendage Ligation*") OR TITLE-ABS-KEY("LA Appendage Ligation*") OR TITLE-ABS-KEY("LAA Ligation*") OR TITLE-ABS-KEY("LAAL*") OR TITLE-ABS-KEY("Watchman Device*") OR TITLE-ABS-KEY("Watchman*") OR TITLE-ABS-KEY("Watchman FLX*") OR TITLE-ABS-KEY("Watchman 2.5*") OR TITLE-ABS-KEY("Amplatzer Amulet*") OR TITLE-ABS-KEY("Amplatzer*") OR TITLE-ABS-KEY("LARIAT Suture*") OR TITLE-ABS-KEY("CLOSURE Device*") OR TITLE-ABS-KEY("LAA Closure Device*") OR TITLE-ABS-KEY("ACURATE LAA*") OR TITLE-ABS-KEY("Atriclip*"))
**Cochrane Library**
ID Search Hits<br>#1 MeSH descriptor: [Atrial Fibrillation] explode all trees 7315<br>#2 (atrial fibrillation):ti,ab,kw (Word variations have been searched) 17106<br>#3 (auricular fibrillation):ti,ab,kw (Word variations have been searched) 43<br>#4 MeSH descriptor: [Left Atrial Appendage Closure] explode all trees 3<br>#5 MeSH descriptor: [Left Atrial Appendage Closure] explode all trees 3<br>#6 (Left atrial appendage closure):ti,ab,kw (Word variations have been searched) 283<br>#7 (#1 OR #2 OR #3) AND (#4 OR #5 OR #6) 232

Table S.3. Approaches to data extraction issues

Issue	Approach
Demographic data were separately reported for various subgroups of one particular group of interest	Groups were merged into a single group using the suggested Cochrane formula [1]
Continuous variables were presented as median (IQR)	Medians and IQR were converted to mean (SD) [2,3]

Abbreviations: IQR, interquartile range; SD, standard deviation

Table S.4. Table of excluded studies with rationale.

Reason for exclusion (Number)	References of excluded studies
Case report (n = 1)	[4]
Narrative review (n = 1)	[5]
Irrelevant population (n = 1)	[6]
Not examining combined M-TEER/LAAO intervention (n=5)	[7–11]
Not reporting outcomes specifically for the combined M-TEER/LAAO (n=1)	[12]
Letter/Comment (n = 2)	[13,14]

Abbreviations: M-TEER, mitral transcatheter edge-to-edge repair; LAAO, left atrial appendage occlusion

Table S.5. Study quality assessment with ROBINS-I

First author, year	Domain 1. Bias due to Confounding	Domain 2. Bias in classification of interventions	Domain 3. Bias in Selection of Participants	Domain 4. Bias due to deviations from intended interventions	Domain 5. Bias due to Missing Data	Domain 6. Bias arising from Measurement of Outcome	Domain 7. Bias in Selection of the Reported Result	Overall Risk of Bias
Kuwata S, 2017 [15]	Moderate	Low	Moderate	Low	Low	Low	Low	Moderate
Frazzetto M, 2023 [16]	Low	Low	Low	Low	Low	Low	Low	Low

Abbreviations: ROBINS-I, Risk of Bias in Non-Randomized Studies of Interventions

Table S.6. Subgroup analysis based on study type

Outcome	Observational studies (n)	ES* with 95%CI (observational studies)	I^2 (observational studies)	Case series (n)	ES* with 95%CI (case series)	I^2 (case series)	$p_{subgroup}$
**A. Dichotomous outcomes**							
All-cause death	4	0.13 (0.07, 0.23)	0	1	0.33 (0.03, 3.2)	N/A	0.417
Any procedural complication	2	0.08 (0.02, 0.26)	0	1	0.09 (0.01, 1.64)	N/A	0.935
Bleeding	5	0.08 (0.02, 0.26)	0.59	1	0.09 (0.01, 1.64)	N/A	0.918
Device thrombosis	1	0.01 (0, 0.2)	N/A	2	0.1 (0.01, 0.79)	0	0.23
Embolism	3	0.02 (0, 0.1)	0	2	0.2 (0.03, 1.21)	0	0.061
Myocardial Infarction	1	0.02 (0, 0.27)	N/A	1	0.11 (0.01, 2.06)	N/A	0.354
Post-procedure MR>2+	2	0.2 (0.01, 2.85)	0.84	2	0.1 (0.01, 0.79)	0	0.688
Procedural success	5	8.72 (2.15, 35.39)	0.82	2	9.96 (1.27, 77.86)	0	0.917
Residual leak	3	0.03 (0.01, 0.13)	0	1	0.09 (0.01, 1.64)	N/A	0.517
Stroke	5	0.03 (0.01, 0.09)	0	1	0.11 (0.01, 2.06)	N/A	0.42
Tamponade	4	0.03 (0.01, 0.09)	0	1	0.11 (0.01, 2.06)	N/A	0.374
**B. Continuous outcomes**							
Contrast (ml)	4	83.27 (59.33, 107.21)	0.83	1	125 (76, 174)	N/A	0.134
Length of stay (days)	3	5.25 (2.82, 7.67)	0.85	1	5 (1.57, 8.43)	N/A	0.908
Number of clips	4	1.64 (1.26, 2.02)	0.94	1	1.6 (0.82, 2.38)	N/A	0.922
Procedural time (min)	5	99.52 (78.59, 120.44)	0.93	2	112.91 (96.89, 128.94)	0	0.319
Radiation time (min)	5	31.76 (24.87, 38.64)	0.92	2	24.13 (13.18, 35.07)	0.55	0.248

*Effect estimates (ES) are presented as risk ratios for dichotomous outcomes and mean differences for continuous outcomes. Abbreviations: ES, effect estimate; MR, mitral regurgitation;

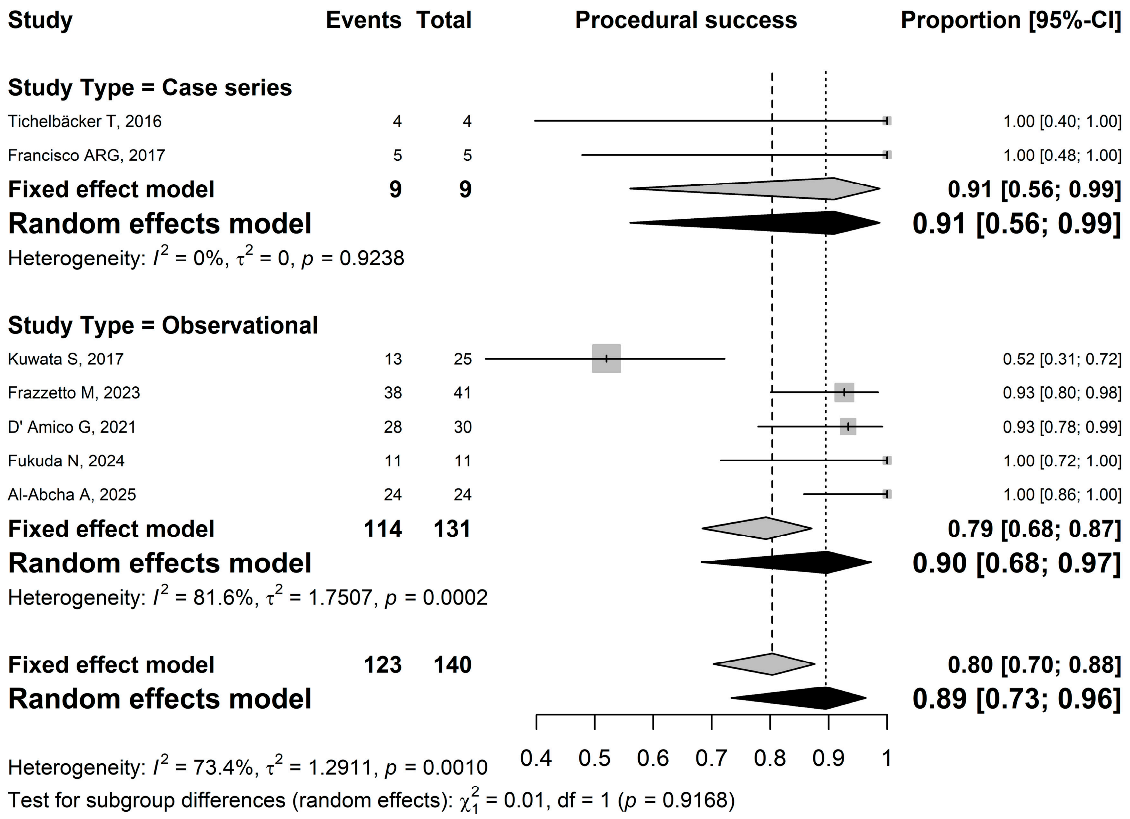

Figure S.1. Forest plot for the outcome of procedural success (M-TEER/LAAO only). CI, confidence interval

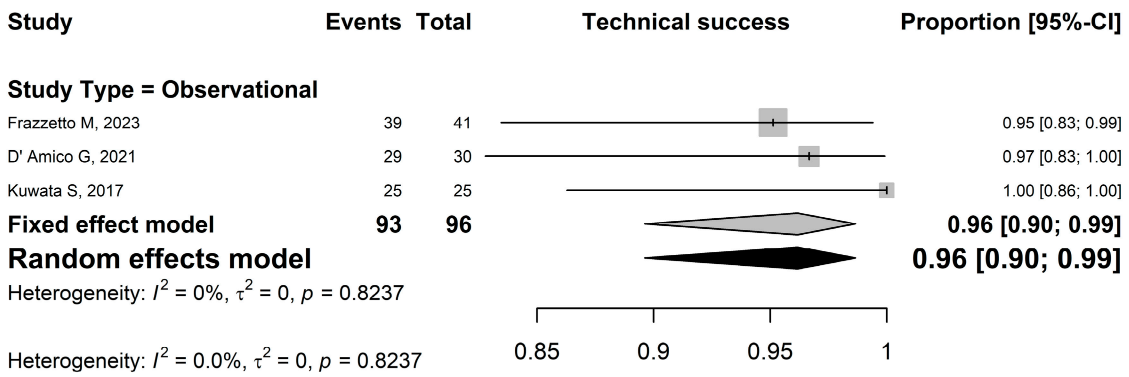

Figure S.2. Forest plot for the outcome of technical success (M-TEER/LAAO only). CI, confidence interval

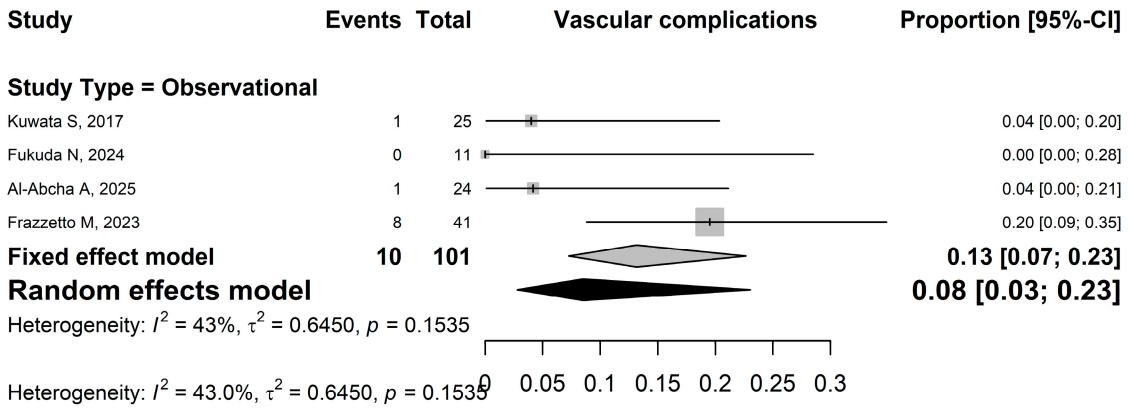

Figure S.3. Forest plot for the outcome of vascular complications (M-TEER/LAAO only). CI, confidence interval

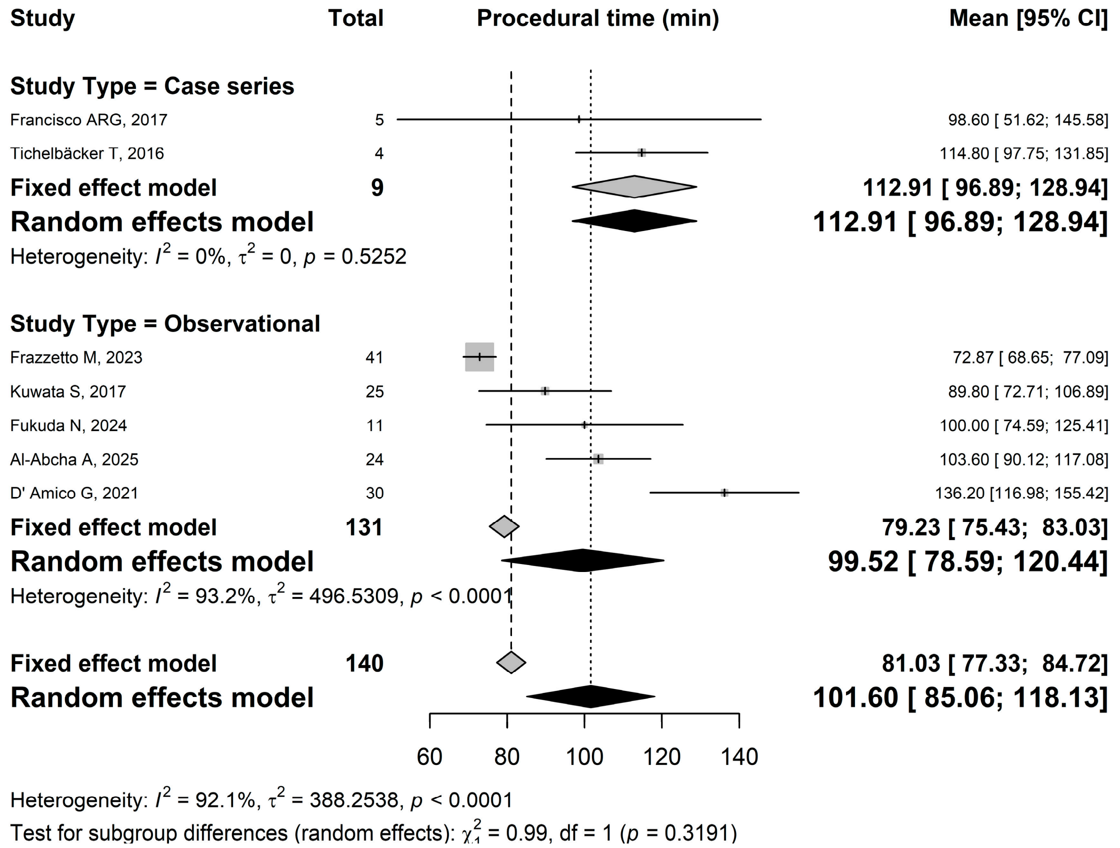

Figure S.4. Forest plot for the outcome of procedural time (M-TEER/LAAO only). CI, confidence interval

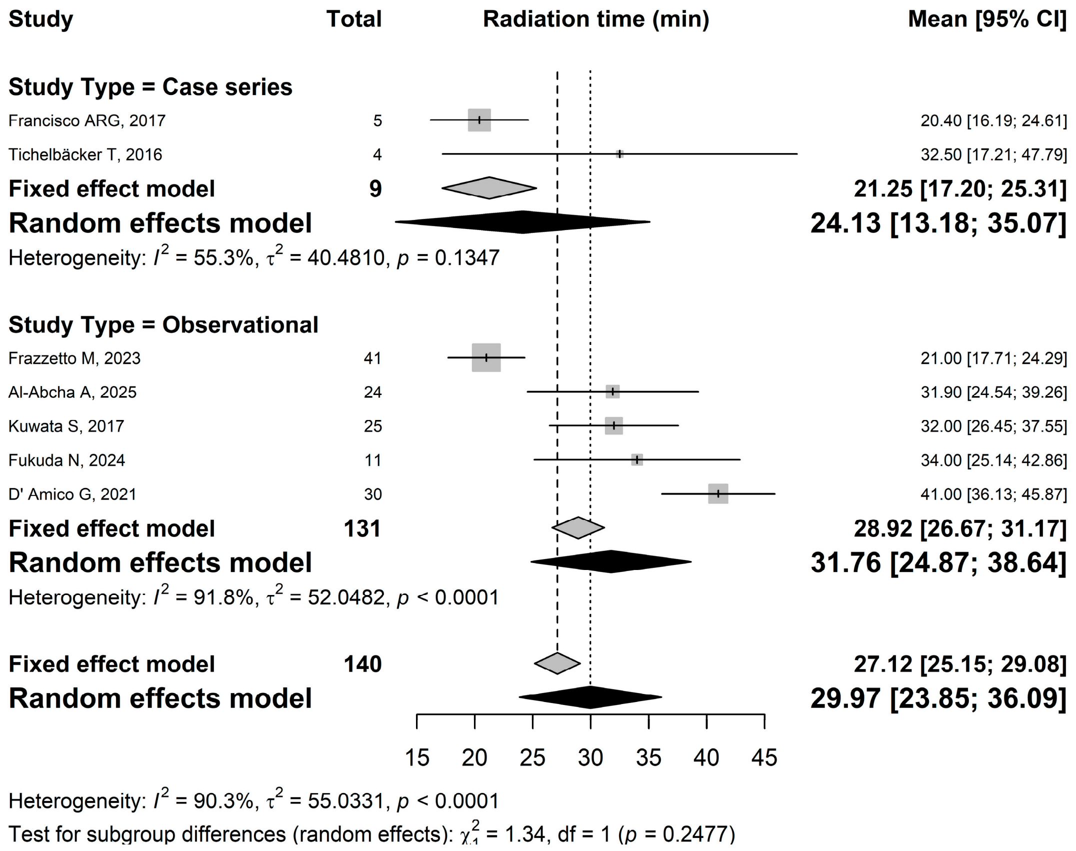

Figure S.5. Forest plot for the outcome of radiation time (M-TEER/LAAO only). CI, confidence interval

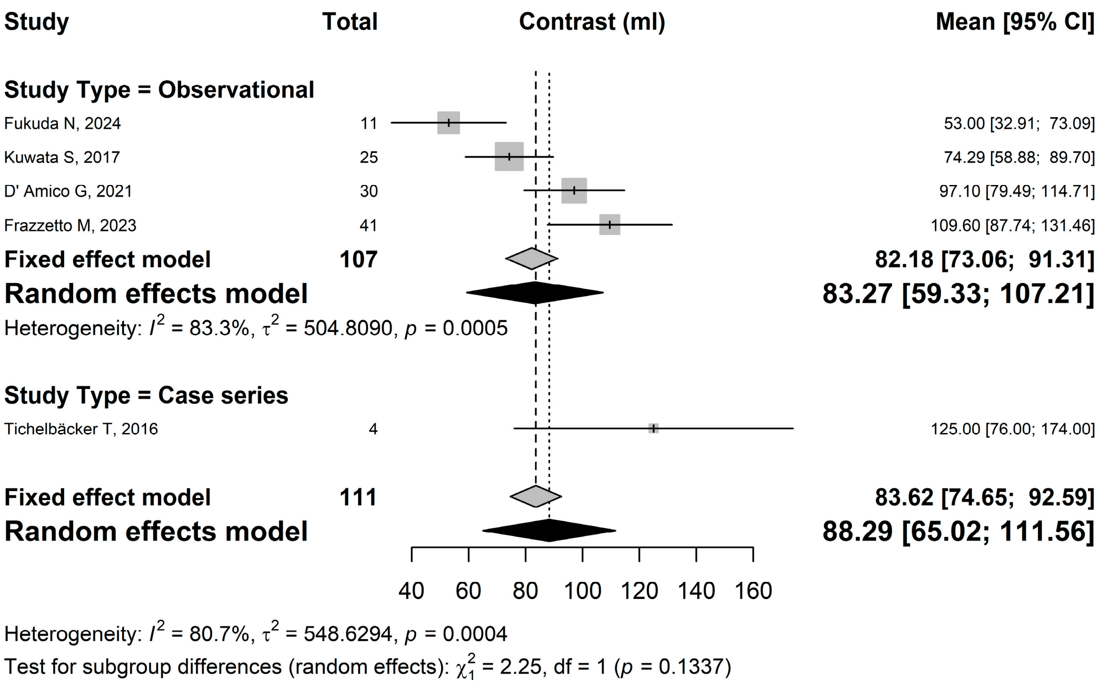

Figure S.6. Forest plot for the outcome of administered contrast (M-TEER/LAAO only). CI, confidence interval

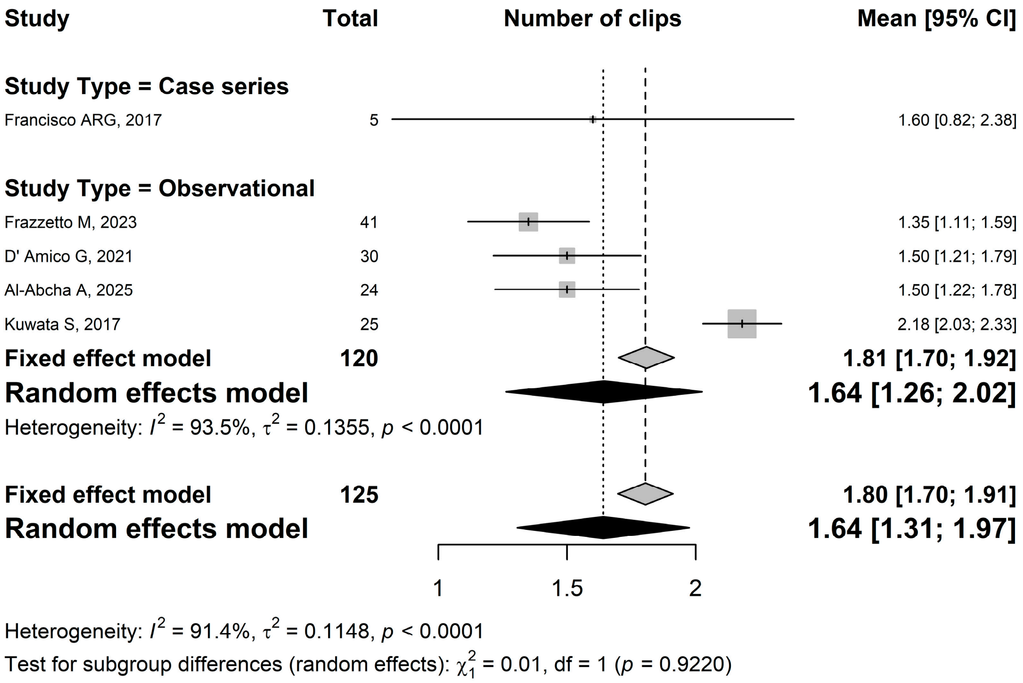

Figure S.7. Forest plot for the outcome of the number of implanted clips (M-TEER/LAAO only). CI, confidence interval

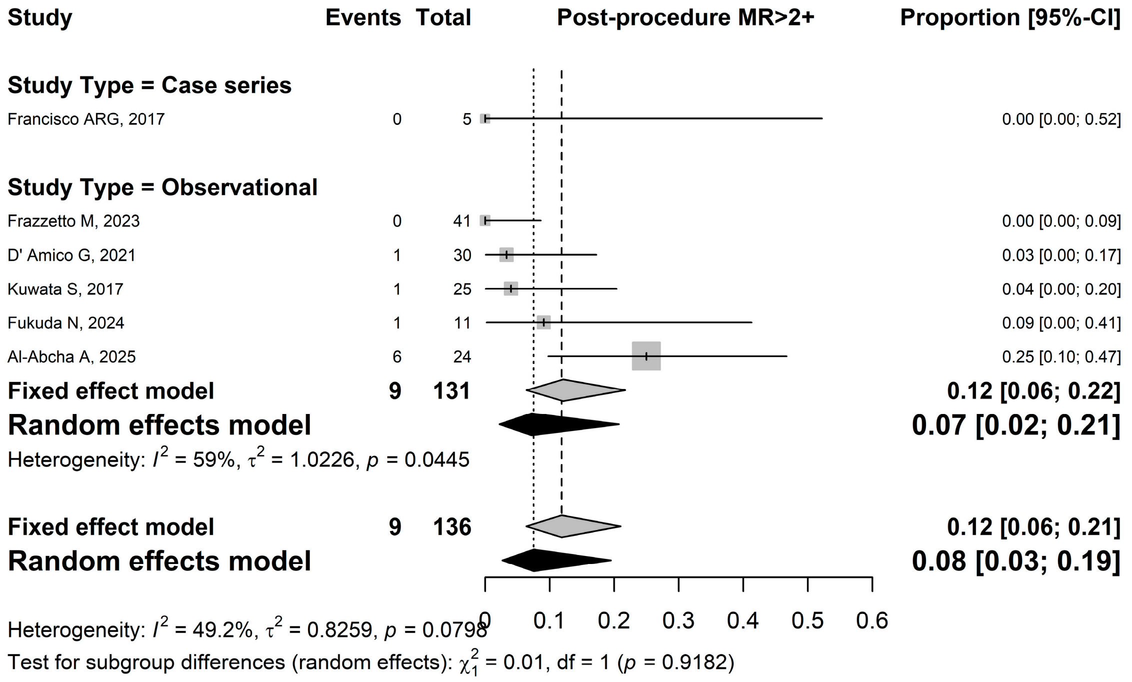

Figure S.8. Forest plot for the outcome of post-procedure MR>2+ (M-TEER/LAAO only). CI, confidence interval; MR, mitral regurgitation.

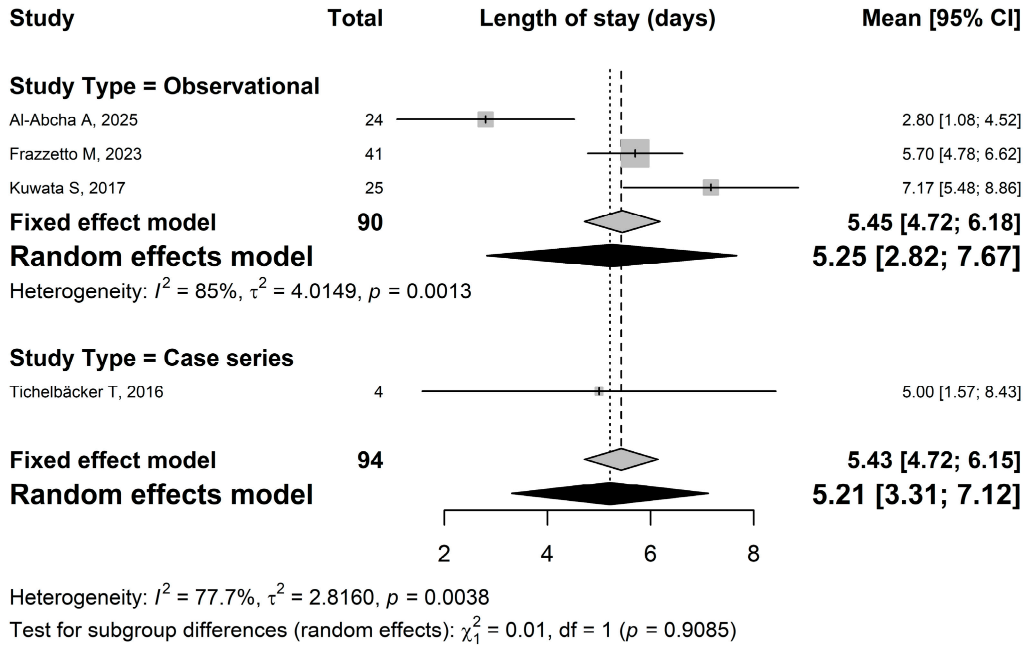

Figure S.9. Forest plot for the outcome of the duration of length of stay (M-TEER/LAAO only). CI, confidence interval

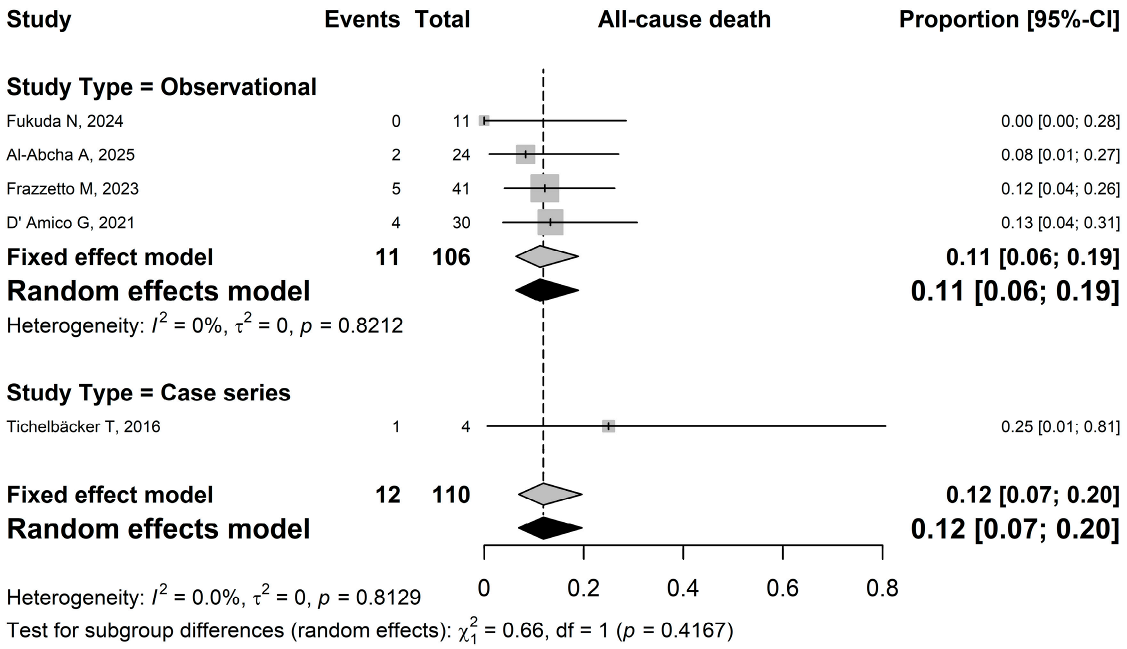

Figure S.10. Forest plot for the outcome of all-cause death (M-TEER/LAAO only). CI, confidence interval

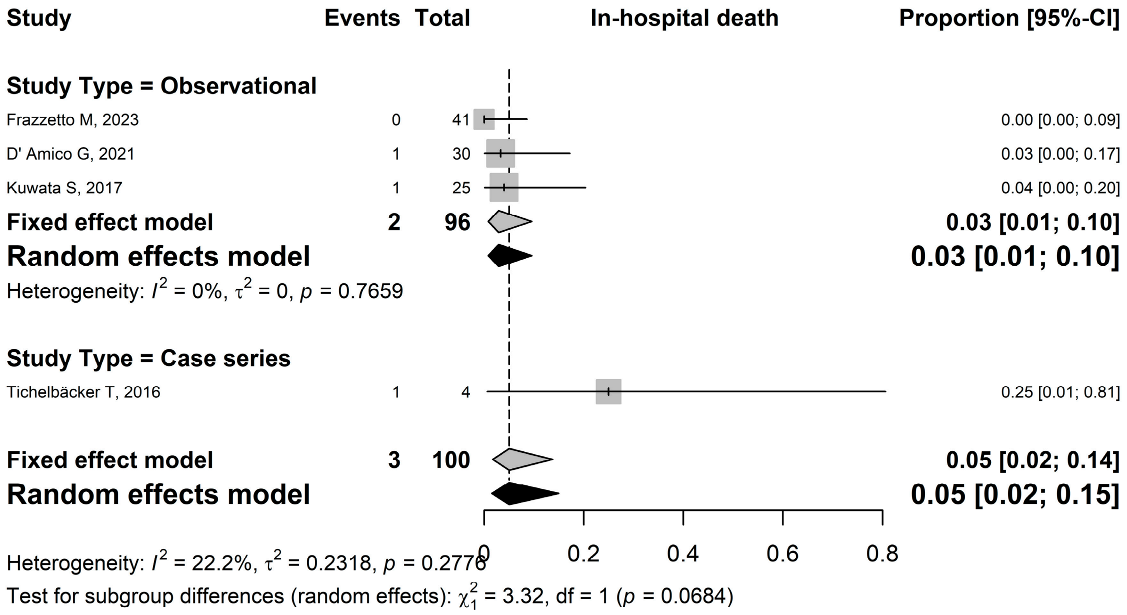

Figure S.11. Forest plot for the outcome of in-hospital death (M-TEER/LAAO only). CI, confidence interval

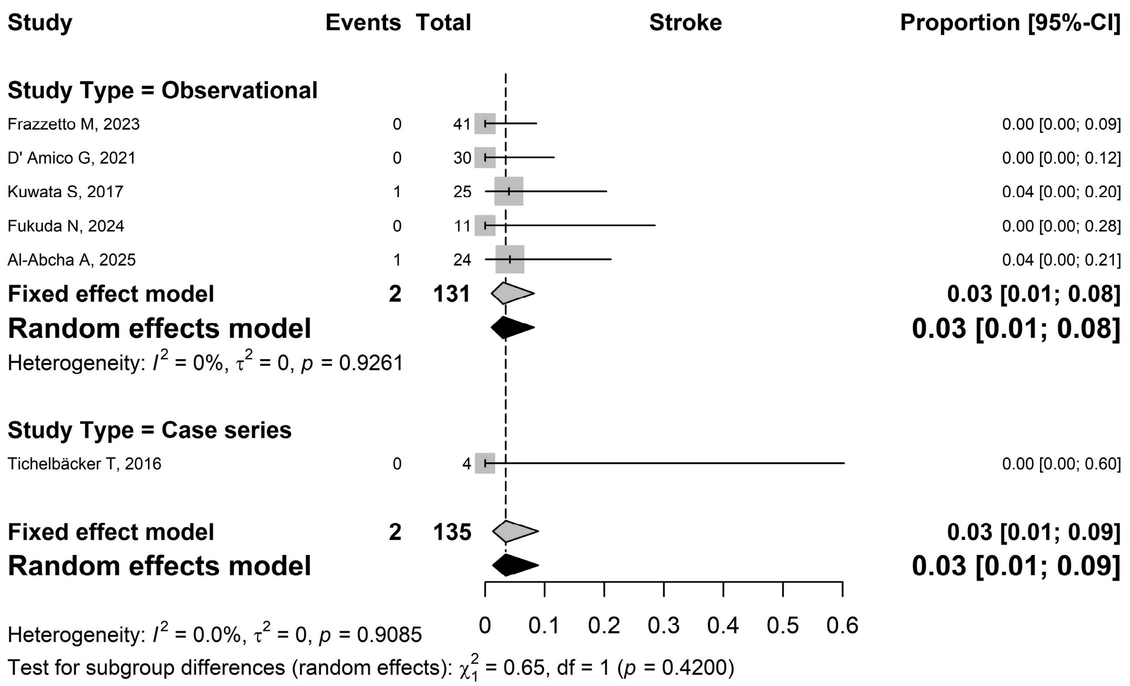

Figure S.12. Forest plot for the outcome of stroke (M-TEER/LAAO only). CI, confidence interval

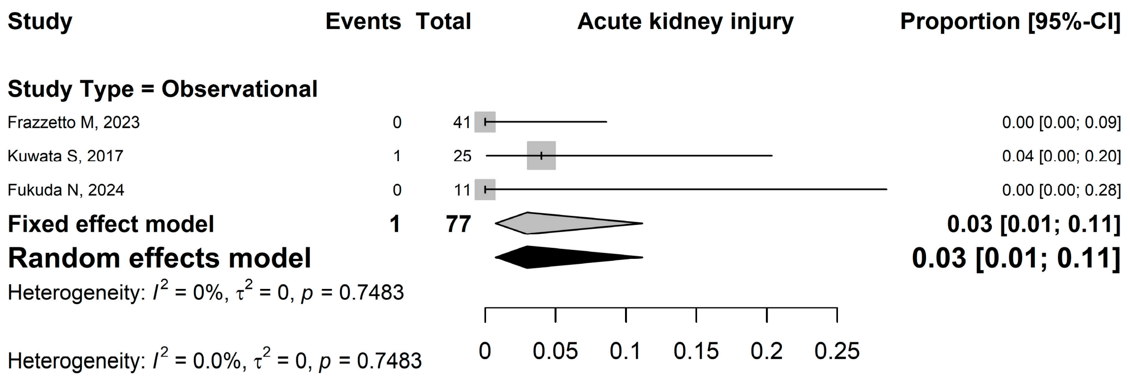

Figure S.13. Forest plot for the outcome of acute kidney injury (M-TEER/LAAO only). CI, confidence interval

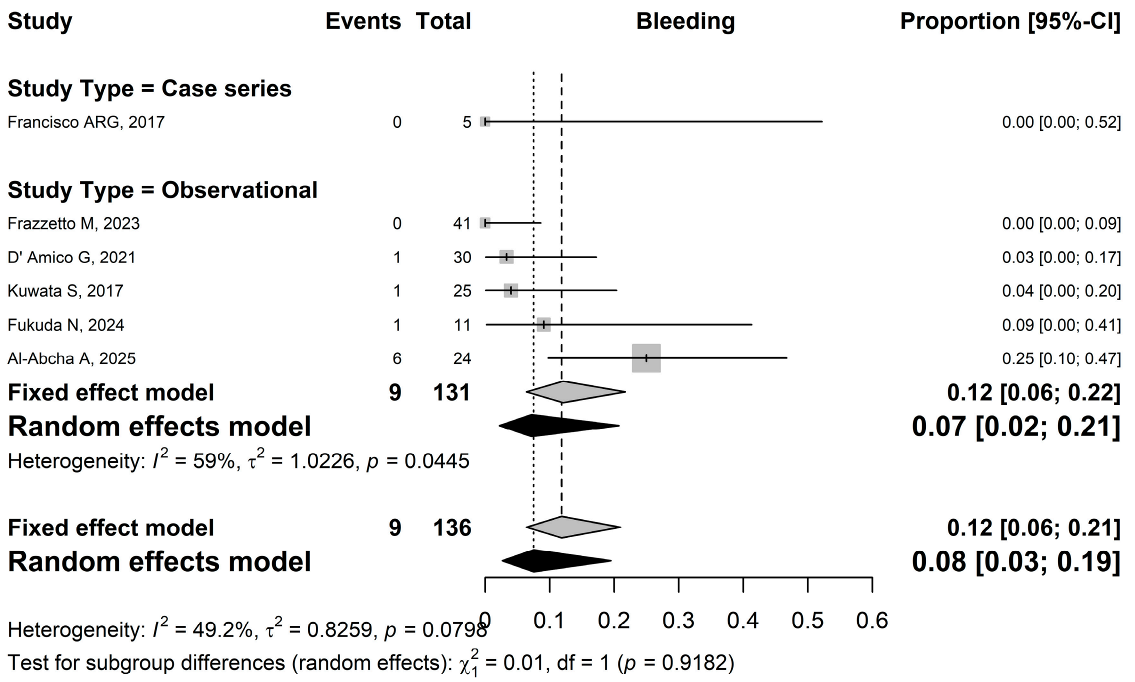

Figure S.14. Forest plot for the outcome of bleeding (M-TEER/LAAO only). CI, confidence interval

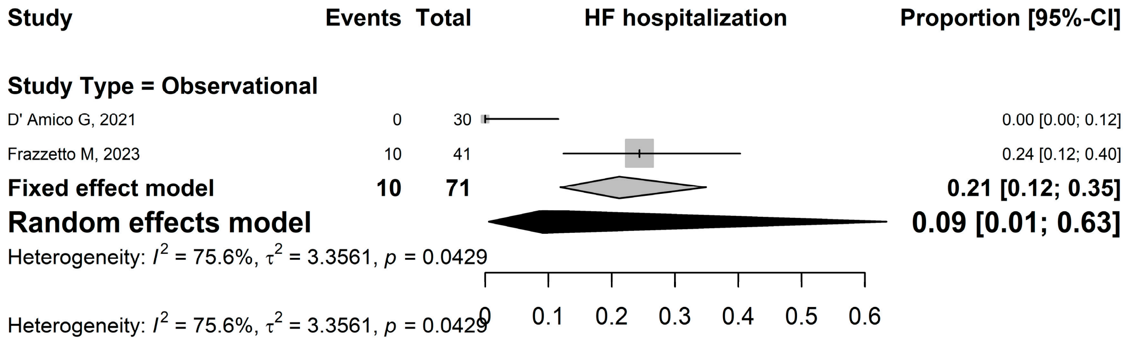

Figure S.15. Forest plot for the outcome of HF hospitalization (M-TEER/LAAO only). CI, confidence interval

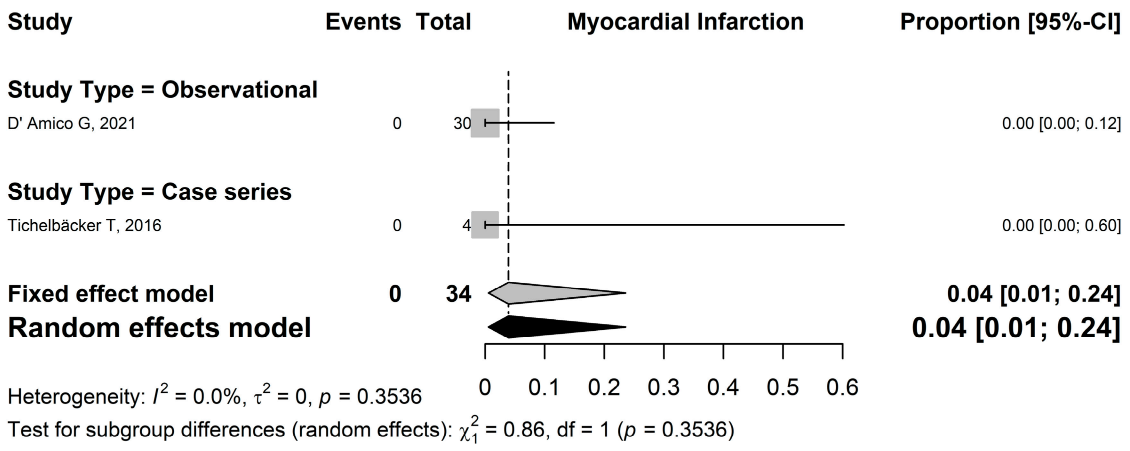

Figure S.16. Forest plot for the outcome of myocardial infarction (M-TEER/LAAO only). CI, confidence interval

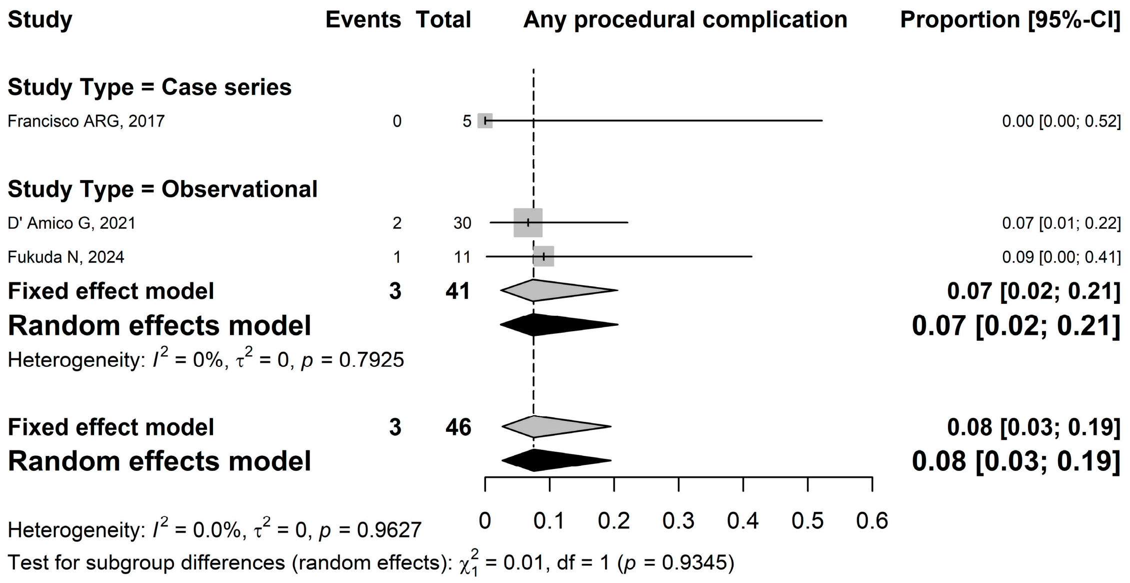

Figure S.17. Forest plot for the outcome of any procedural complication (M-TEER/LAAO only). CI, confidence interval

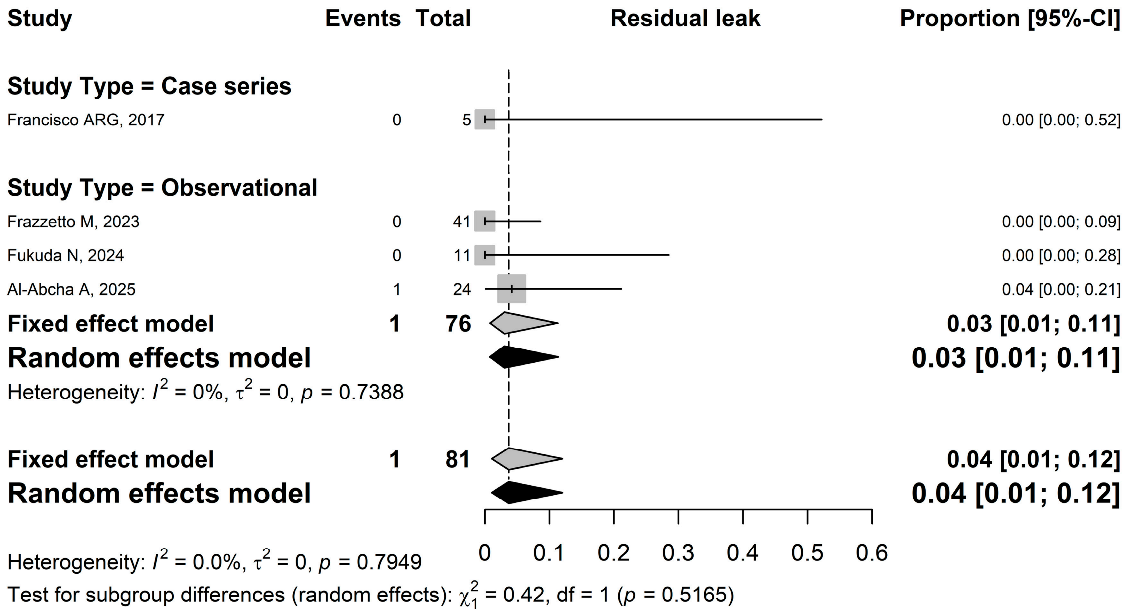

Figure S.18. Forest plot for the outcome of residual LAAO leak (M-TEER/LAAO only). CI, confidence interval

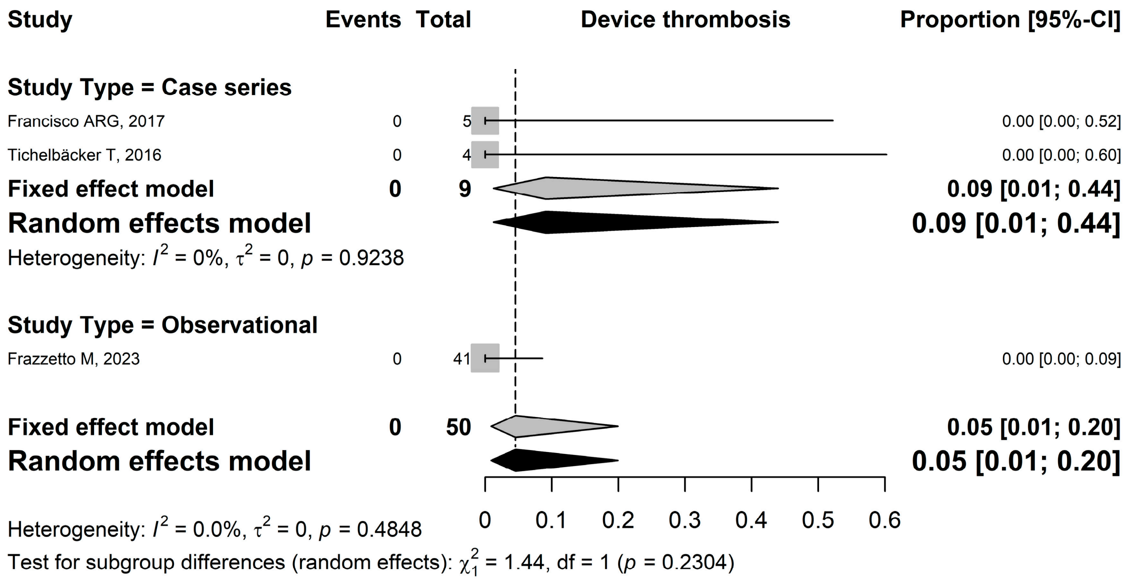

Figure S.19. Forest plot for the outcome of device thrombosis (M-TEER/LAAO only). CI, confidence interval

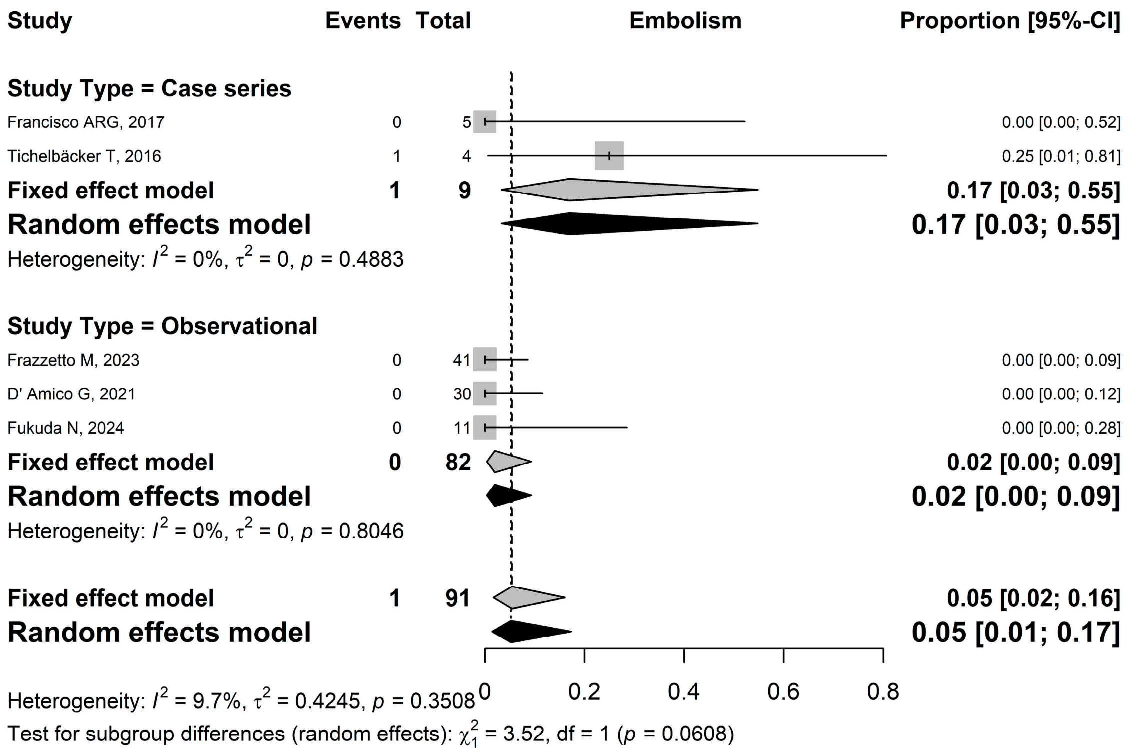

Figure S.20. Forest plot for the outcome of embolism (M-TEER/LAAO only). CI, confidence interval

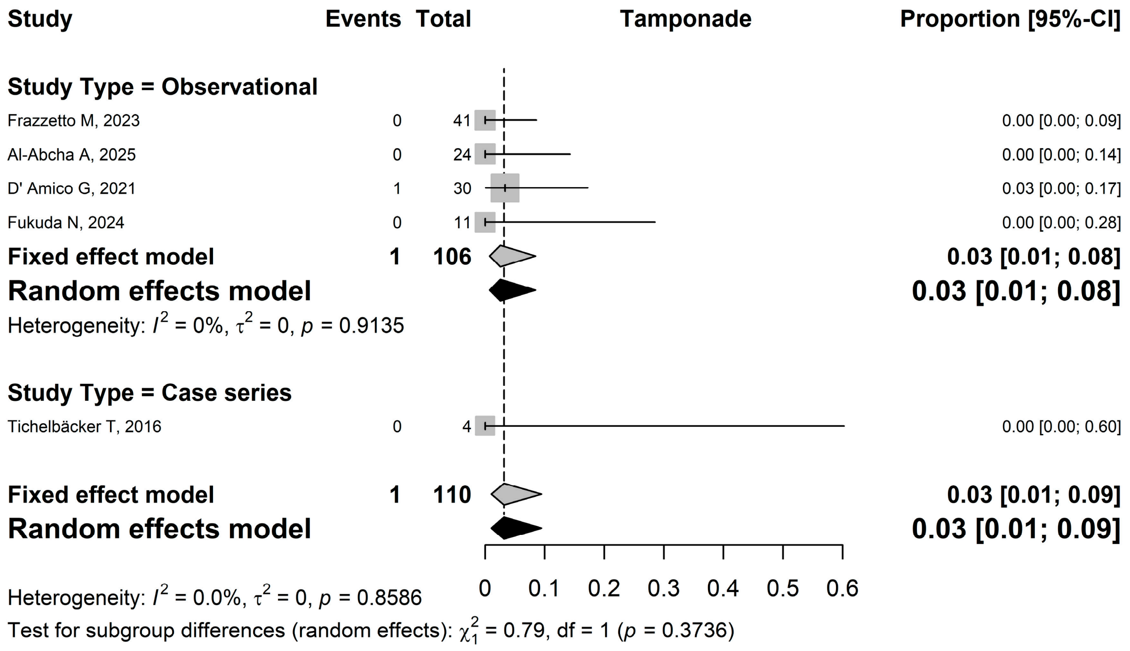

Figure S.21. Forest plot for the outcome of tamponade (M-TEER/LAAO only). CI, confidence interval

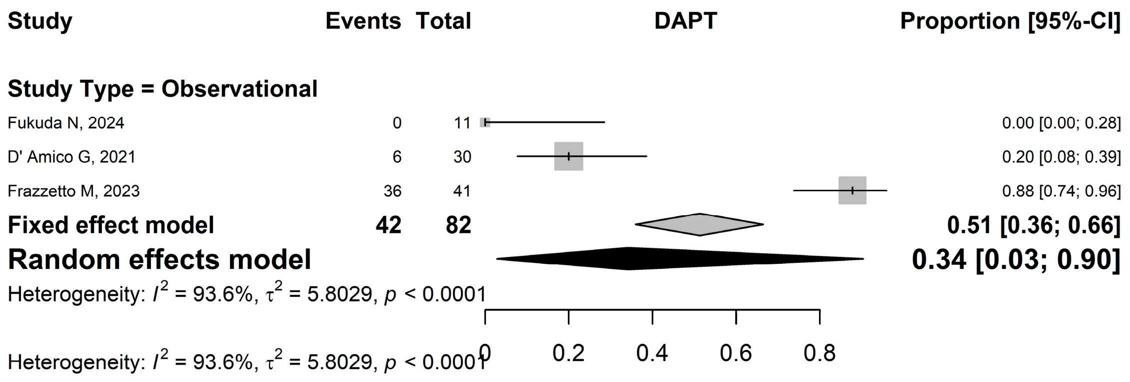

Figure S.22. Forest plot for the outcome of discharge on dual antiplatelet therapy (DAPT) (M-TEER/LAAO only). CI, confidence interval

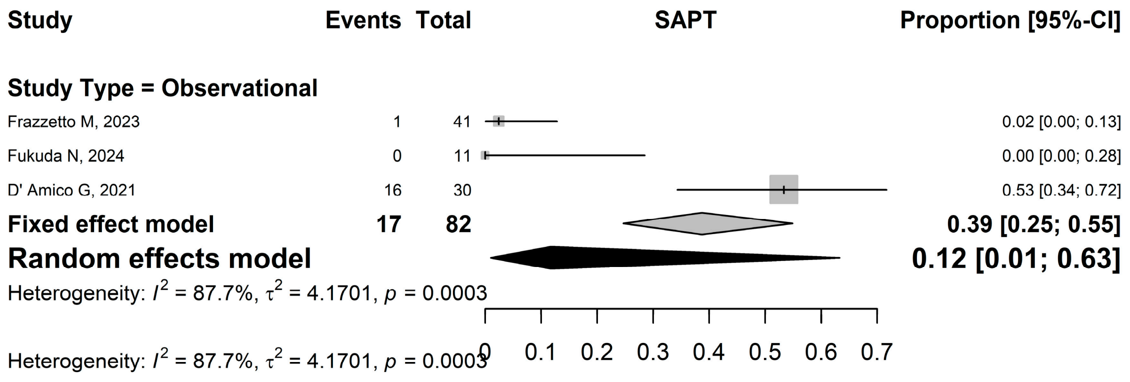

Figure S.23. Forest plot for the outcome of discharge on single antiplatelet therapy (SAPT) (M-TEER/LAAO only). CI, confidence interval

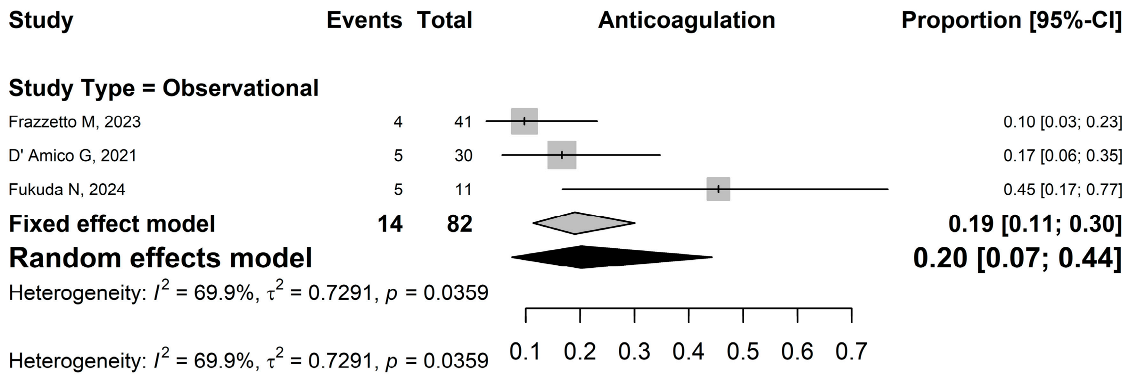

Figure S.24. Forest plot for the outcome of discharge on anticoagulation (M-TEER/LAAO only). CI, confidence interval

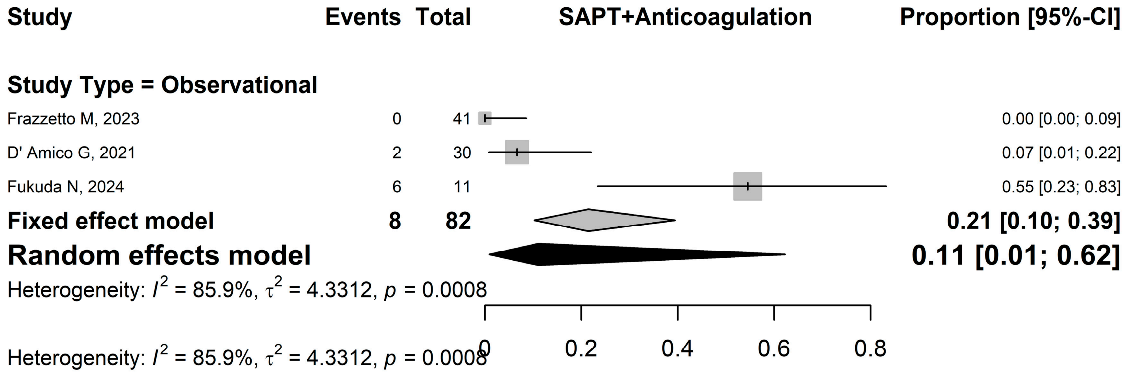

Figure S.22. Forest plot for the outcome of discharge on single antiplatelet therapy (SAPT) and anticoagulation (M-TEER/LAAO only). CI, confidence interval

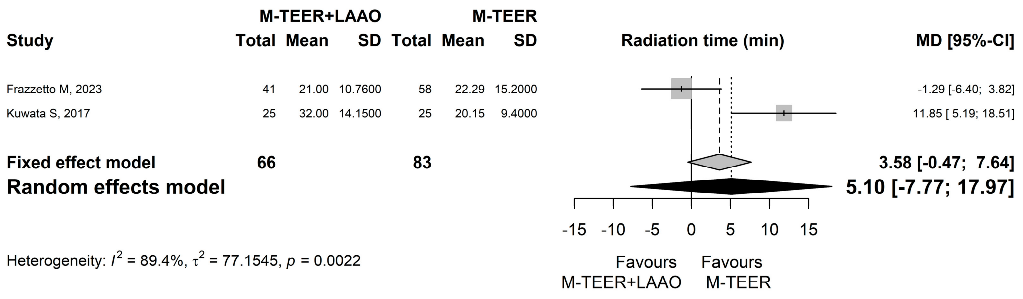

Figure S.23. Forest plot for the outcome of radiation time (M-TEER/LAAO vs M-TEER). MD, mean difference; SD, standard deviation; CI, confidence interval; M-TEER, mitral transcatheter edge-to-edge repair; LAAO, left atrial appendage occlusion

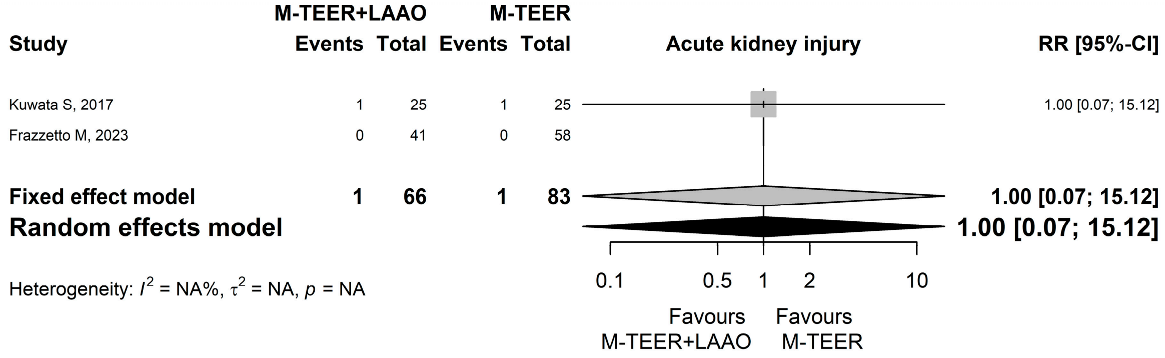

Figure S.24. Forest plot for the outcome of acute kidney injury (M-TEER/LAAO vs M-TEER). RR, risk ratio; CI, confidence interval; M-TEER, mitral transcatheter edge-to-edge repair; LAAO, left atrial appendage occlusion.

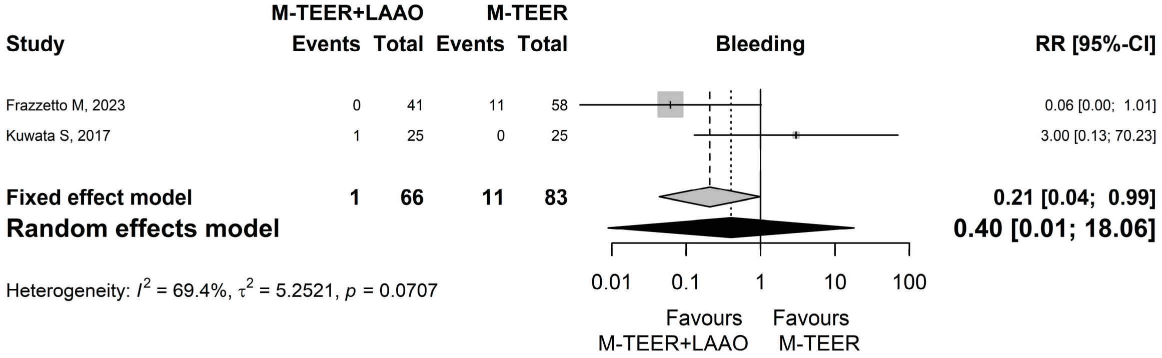

Figure S.25. Forest plot for the outcome of bleeding (M-TEER/LAAO vs M-TEER). RR, risk ratio; CI, confidence interval; M-TEER, mitral transcatheter edge-to-edge repair; LAAO, left atrial appendage occlusion

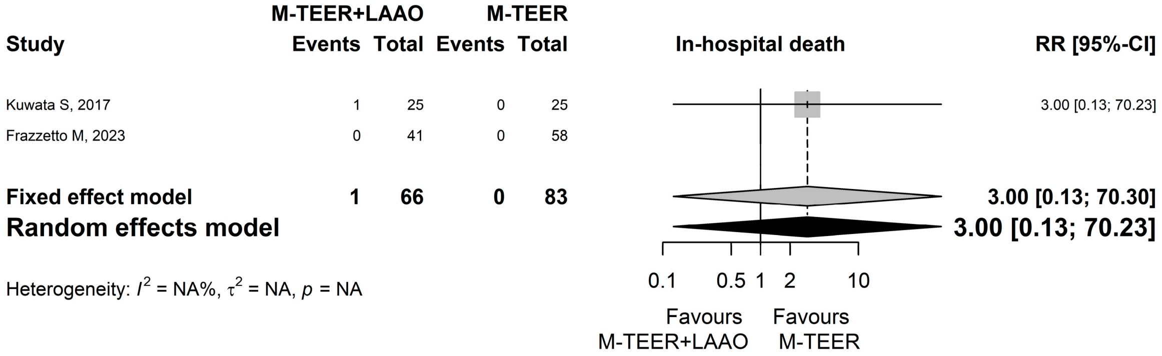

Figure S.26. Forest plot for the outcome of in-hospital death (M-TEER/LAAO vs M-TEER). RR, risk ratio; CI, confidence interval; M-TEER, mitral transcatheter edge-to-edge repair; LAAO, left atrial appendage occlusion

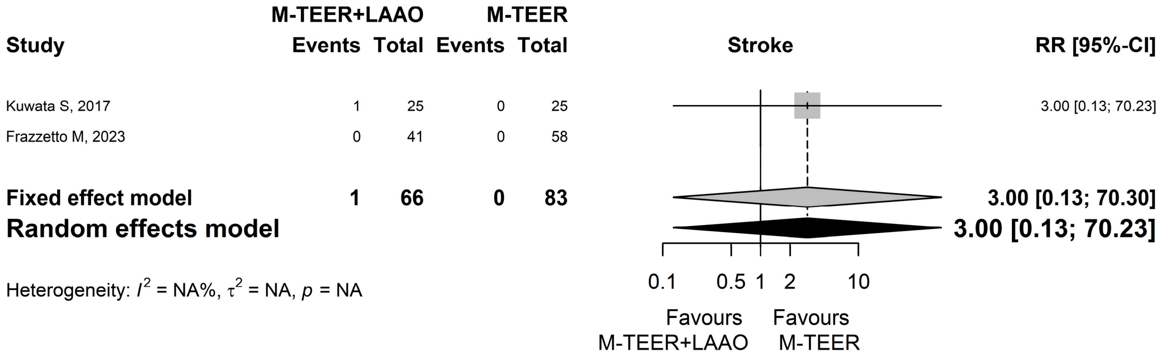

Figure S.27. Forest plot for the outcome of stroke (M-TEER/LAAO vs M-TEER). RR, risk ratio; CI, confidence interval; M-TEER, mitral transcatheter edge-to-edge repair; LAAO, left atrial appendage occlusion
